# Learning-induced reorganization of number neurons and emergence of numerical representations in a biologically inspired neural network

**DOI:** 10.1038/s41467-023-39548-5

**Published:** 2023-06-29

**Authors:** Percy K. Mistry, Anthony Strock, Ruizhe Liu, Griffin Young, Vinod Menon

**Affiliations:** 1 Department of Psychiatry & Behavioral Sciences, Stanford University School of Medicine, Stanford, CA 94304 USA; 2 Department of Neurology & Neurological Sciences, Stanford University School of Medicine, Stanford, CA 94304 USA; 3 Wu Tsai Stanford Neuroscience Institute, Stanford University School of Medicine, Stanford, CA 94304 USA; 4 Graduate School of Education, Stanford University, Stanford, CA 94304 USA; 5 Stanford Institute for Human-Centered AI, Stanford University, Stanford, CA 94304 USA

**Keywords:** Cognitive neuroscience, Computational models, Learning and memory

## Abstract

Number sense, the ability to decipher quantity, forms the foundation for mathematical cognition. How number sense emerges with learning is, however, not known. Here we use a biologically-inspired neural architecture comprising cortical layers V1, V2, V3, and intraparietal sulcus (IPS) to investigate how neural representations change with numerosity training. Learning dramatically reorganized neuronal tuning properties at both the single unit and population levels, resulting in the emergence of sharply-tuned representations of numerosity in the IPS layer. Ablation analysis revealed that spontaneous number neurons observed prior to learning were not critical to formation of number representations post-learning. Crucially, multidimensional scaling of population responses revealed the emergence of absolute and relative magnitude representations of quantity, including mid-point anchoring. These learnt representations may underlie changes from logarithmic to cyclic and linear mental number lines that are characteristic of number sense development in humans. Our findings elucidate mechanisms by which learning builds novel representations supporting number sense.

## Abstract (web summary)

How the brain represents numbers remains poorly understood. Here, the authors uncover the emergence of absolute and relative magnitude representations of quantity in a biologically-inspired neural network, mirroring observations in children during numerical skill acquisition.

## Introduction

How the nervous system represents and categorizes numerical quantity and numbers is poorly understood. Human neuroimaging studies have shown that numerical quantity is represented by distributed patterns of neural activity^1–3^. In contrast, electrophysiological recordings in non-human primate brains, and to a limited extent in the human brain, have reported number-sensitive neurons which respond preferentially to a specific number of objects. For example, a neuron preferring numerosity “5” has the highest average firing rate when five objects are presented to the animal, and progressively lower ones for other numerosities^4,5^. Precisely how numerical representations emerge in the human brain is poorly understood, and acquiring intracranial electrophysiological data in children during development for addressing this challenge is largely implausible at this time. In-silico experiments with biologically inspired neural networks provide an alternative and computationally rigorous approach for examining how neuronal representations develop with learning. Here, we use a biologically-inspired deep neural network to investigate how neural coding of quantity emerges with learning, at both the single unit and distributed population levels.

In the first study of its kind, Stoianov and Zorki^6^ showed that visual numerosity can emerge as a statistical property of images in deep neural networks (DNN) that learn a hierarchical generative model of sensory input. A deep belief network was trained to reconstruct an image of 1 to 32 white rectangles on a black background. Number-tuning neurons with monotonic responses to numerosity emerged in the second hidden layer of the network, despite the learning goal being number-irrelevant. Extending this work, recent studies have shown that quantity sensitive neurons can emerge spontaneously in convolutional neural networks trained to categorize objects in standardized LSVRC-2012 ImageNet datasets^7^. Although the network was not trained on categorization of numerical quantity, units in the final network layer were selective for numerosity. The emergence of spontaneous number neurons (SPONs) in the absence of explicit numerosity training was posited to underlie number sense. Subsequently, Kim, et al.^8^ showed that neurons with similar response properties can also appear in randomly intitialized networks. Contrary to Nasr, et al.^7^, they proposed that visual object training was not relevant for the initial emergence of numerosity. Finally, Zhang & Wu^9^ identified potential methdological issues associated with identification of SPONs in prior studies.

While these studies shed light on how precursors of number sense might emerge in an artificial neural network, they do not directly address how tuning of neural responses and distributed population representations are altered by learning and development. Moreover, numerosity tuning of neurons in these studies was assessed indirectly, using a mutistep process involving either a second neural network^7^ or a support vector machine^8^ to learn number comparison, without altering the pre-trained network and the underlying neural representations of numerosity. Thus, it is not known whether SPONs and distributed neural representations remain stable or whether they get reorganized with end-to-end learning. Importantly, neither study directly probed mapping between non-symbolic and symbolic representations of quantity, which is one possible account of the emergence of number sense in humans^2,10^.

In this study, we adopt an approach to DNN-based learning that is developmentally informed. The acquisition of numerical skills in children is often described by the mapping account, which suggests that linking non-symbolic representations of quantities to abstract symbolic representations is critical for the development of numerical problem-solving abilities^11–15^. However, alternative theoretical views propose that the ‘symbol-grounding’ problem for symbolic numbers remains unresolved^16,17^, and that number sense and symbolic number processing capabilities may rely on separate neural systems^18^. Despite these theoretical differences, there is a consensus that children initially learn the meaning of small numbers by mapping them to non-symbolic representations, while larger numbers may be acquired through counting and arithmetic principles^19,20^. Brain imaging studies suggest that stronger neural representational similarity between symbolic and non-symbolic representations during early development is associated with better mathematical problem-solving skills^2,10^ but this mapping weakens as skills mature^10^. Thus, learning to map symbolic representations of quantity with non-symbolic referents or visual primitives provides an ideal platform for an in-silico investigation of plausible neuronal and population-level mechanisms underlying early numerical skill acquisition. Our approach aligns with the view that exact numerical representations are not innately predetermined but rather are learned through enculturation^21,22^.

**Fig. 1:**
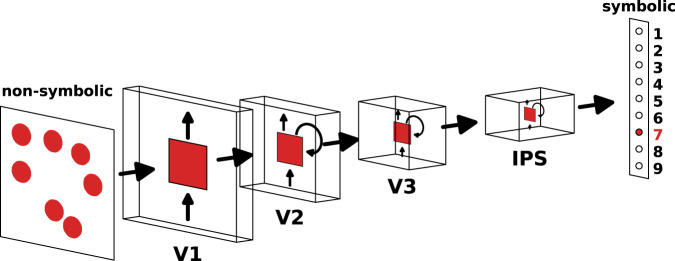
Architecture of number deep neural network (nDNN) adapted from the biologically-inspired CORnet-S. nDNN consists of four layers that model hierarchy and recurrent circuit dynamics in areas V1, V2, V3, and IPS of the dorsal visual processing stream. The architecture of nDNN is adapted from CORnet-S, a biologically inspired network architecture for visual object categorization. The nDNN is trained to map non-symbolic representation of numbers to their symbolic representation. The nDNN includes feedforward and recurrent (shown by looped arrows within a layer in the figure) connections.

We implemented a number-DNN (nDNN) model which learned the mapping of non-symbolic stimuli to quantity representations (numerosity training). In an advance over previous studies, we use a biologically more plausible architecture (Fig. 1), inspired by recent work on visual object categorization^23^. nDNN has several advantages over convolution neural network models such as AlexNet which were primarily developed for computer vision applications^24^. Most importantly, biologically-inspired architectures have been shown to be closely match neural and behavioral measures observed in non-human primates^25^. Our nDNN incorporated layers V1, V2, V3, and IPS which model dorsal visual information processing pathways known to play an essential role in numerical cognition^15,26,27^. We investigated how numerosity training reorganizes neural tuning and population-level distributed network representations. Importantly, the numerosity training involved learning to map non-symbolic referents of quantity to abstract symbolic representations of quantity, a skill that is taught to children during early developmental stages^2,28^. Our approach allowed us to capture the emergence of new neuronal-level and population-level representations with training, mimicking the early stages of numbers sense acquisition during development.

**Fig. 2:**
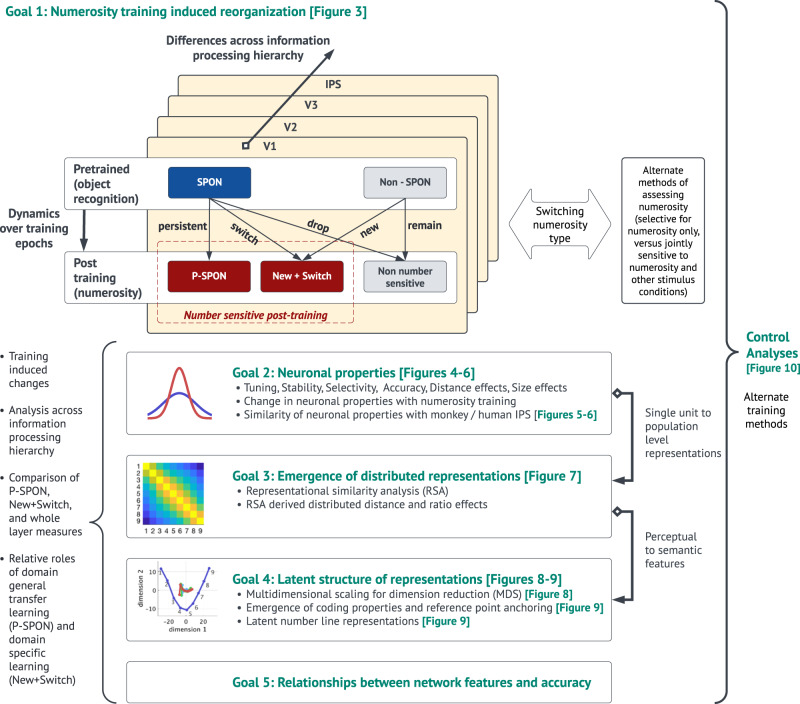
Overview of the key goals and analysis steps. Goal 1 investigated how neurons reorganize with learning and determine whether numerosity training preserves the integrity of spontaneous number neurons (SPONs). Goal 2 examined how learning changes the properties of individual neurons along the information processing hierarchy from V1, V2, and V3 to IPS. We examined several neuronal properties, including neuronal tuning, stability, selectivity, and distance effects at a single unit level, how they change with numerosity training, and how they correspond to neuronal recordings in primate IPS. Goal 3 investigated how distributed population-level representations change with learning. Goal 4 examined latent structure of distributed population-level neural representations, the coding properties that emerge from these representations, and how these coding properties relate to number sense. Goal 5 identified neuronal tuning and distributed population-level representational features that predict network accuracy. In each goal we examined neural reorganization in each nDNN layer along the information processing hierarchy from V1, V2, V3 to IPS. We also performed control analyses using alternate training methods to check the robustness of the findings.

Our study has five main goals, as schematized in Fig. 2. Our first goal was to investigate how neurons reorganize with learning and determine whether numerosity training preserves the integrity of SPONs, neurons that emerge as number sensitive with domain-general image recognition prior to any numerosity training. We examined whether SPONs emerged in the biologically inspired network, and, crucially, quantified their reorganization with numerosity training. Specifically, we tested the hypotheses that a majority of SPONs would not preserve their original preferred numerosity, and that the relative contribution of SPONs to numerosity classification would reduce, after numerosity training. Testing this hypothesis would shed light on the relation between “nativist” perspectives, which posit innate stable neural representations, and “emergentist” perspectives, which posit dynamic representations that evolve with learning^29^. It would also reveal whether SPONs arising from non-numerosity related visual experiences form an essential foundation for numerical cognition, and how numerosity representation depends on neurons that are exclusively selective for numerosity versus those that are sensitive to both numerosity and other factors such as area and size (other stimulus conditions).

The second goal of our study was to investigate how learning changes the properties of individual neurons along the information processing hierarchy from V1, V2, and V3 to IPS. We computed the proportion, acuity, and distribution of number sensitive neurons across different learning epochs. We examined whether training induced fundamental changes in key neuronal properties such as tuning precision, stability, and selectivity of neurons, as well as inferred properties such as numerical distance and size effects. We tested the hypothesis that training would result in the emergence of more precise numerosity tuning in layer IPS of the nDNN, and compared this to number-tuning properties previously observed in monkey and human IPS. A significant change in properties would emphasize the specificity of explicit numerosity training, relative to numerosity that might arise from non-numerosity related visual experiences.

The third goal of our study was to go beyond coding properties of individual number sensitive neurons, and determine how numerosity training leads to the emergence of new distributed population representations across neurons in each layer of the visual information processing hierarchy. To accomplish this we used representational similarity analysis (RSA), similar to procedures used in functional brain imaging studies^30^. We assessed how distributed representations of numerical quantities emerge across the information processing hierarchy, and tested the hypothesis that layer IPS would form distinct patterns encoding numerical representations. We tested whether key distributed representations and canonical observations such as numerical distance and size effects associated with number sensitivity are already present in a network trained only on object perception, or whether additional, number-specific training is necessary for such properties to emerge. We operationalized this by comparing the distributed representations for SPONs that retained their numerosity across training, with those for neurons whose number sensitivity emerged later or switched as a result of training.

The fourth goal of our study was to examine the latent structure of distributed population-level neural representations. We used multidimensional scaling (MDS) to obtain a low-dimensional representation of the neural representational distances between different input numerosities. By mapping stimulus properties against these MDS representations, we were able to identify distinct, cognitively relevant latent dimensions of numerosity, including the reconstruction of latent number line representations. We tested the hypothesis that post-training, distinct latent representations would reflect both absolute magnitude and relative magnitude within the stimuli space, based on distance from anchors, or reference points, analogous to the development of reference points on the number line identified in human behavioral studies^31^.

Our final goal was to identify neuronal tuning and distributed population-level representational features that predict network accuracy. This was accomplished by evaluating the early training periods during which large changes in network accuracy were observed with learning. We related changes in network accuracy to different network properties including the proportion of numerosity sensitive neurons, the ratio of spontaneous and newly formed numerosity neurons, tuning curves, and the MDS-based representational distance, in each network layer.

Our analysis revealed that numerosity training results in dramatic reorganization of both neuronal properties and population-level distributed representations. Furthermore, our analysis uncovered the formation of novel number representations, including absolute and relative magnitude representations of quantity, which were not present prior to numerosity training. Our findings elucidate mechanisms by which learning builds novel representations supporting number sense. A number of control analysis demonstrate the validity and robustness of our findings.

## Results

### Changes in classification accuracy with numerosity training

**Fig. 3:**
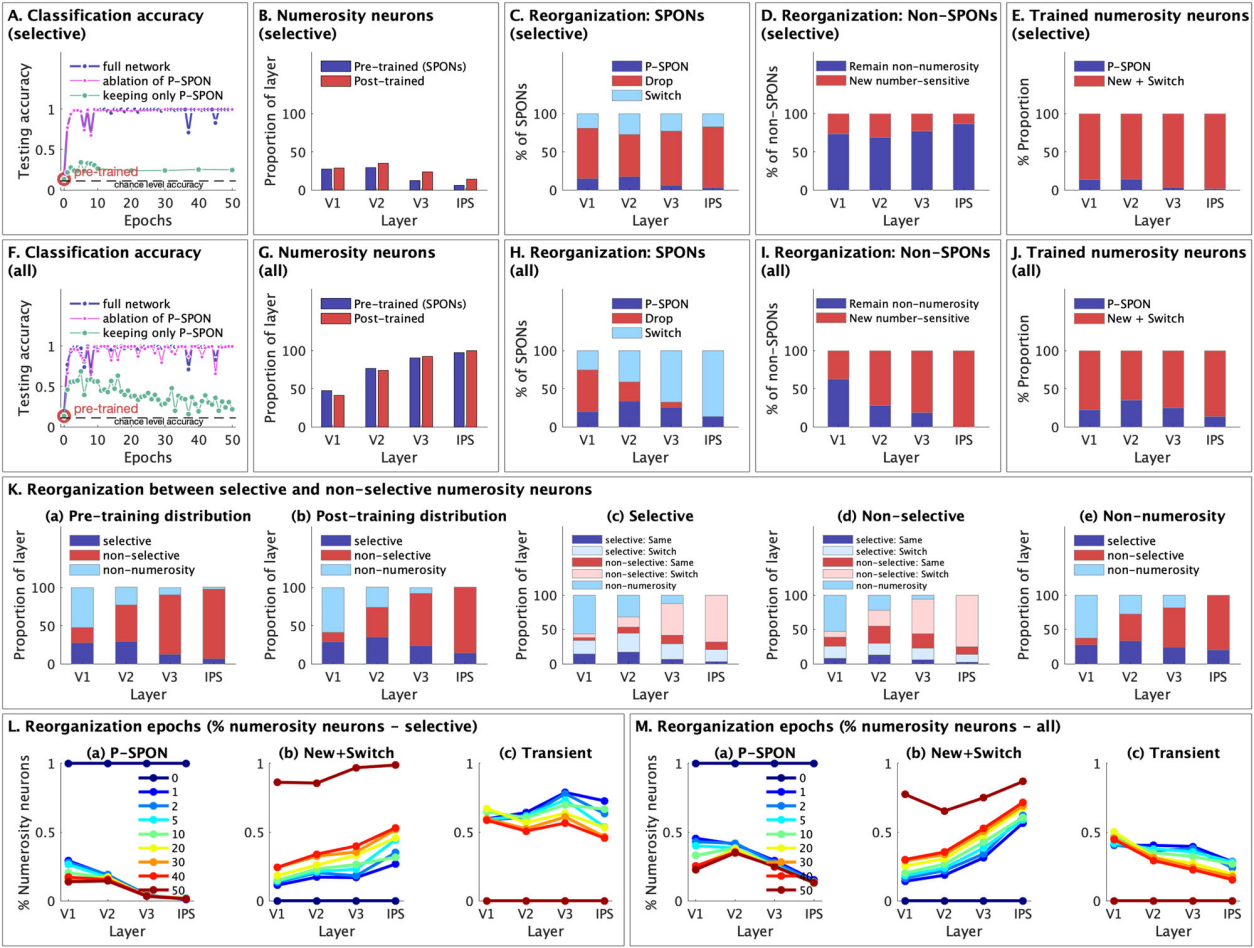
Reorganization of nDNN with numerosity training. (**A**–**E**, **L**: numerosity neurons identified based on being selectively sensitive to numerosity, but not condition; **F**–**J**, **M**: numerosity neurons identified based on being sensitive to numerosity, regardless of whether they are also sensitive to stimulus condition). **A**, **F** Testing accuracy of the pre-trained network and across 50 epochs of numerosity training: full network (blue); network with persistent spontaneous (P-SPON) neurons in IPS layer ablated (pink) does not suffer performance degradation; network with all neurons in IPS layer except P-SPON ablated (green) suffers significant degradation in performance. **B**, **G** Number sensitive neurons as a proportion of total number of neurons in each layer, pre- and post-training. **C**, **H** Reorganization of SPONs with numerosity training: P-SPONs (dark blue), drop number sensitivity (red), or switch numerosity (light blue). **D**, **I** Reorganization of non-SPONs with numerosity training: Proportion of non-SPONs that remain non-sensitive to any number (dark blue), and that change to being number-sensitive (red). **E**, **J** Proportion of number sensitive post training that are newly trained or switched numerosities (dark blue) versus those that are P-SPONs and retain their SPON numerosity (red). **K** Reorganization between numerosity neurons that are exclusively sensitive to numerosity (selective), those that are sensitive to both numerosity and stimulus condition (non-selective), and those that are not sensitive to numerosity. (a, b) show the distribution of these neurons in each layer pre and post training. (c) Shows how the selective numerosity neurons reorganize post-training, with switch indicating a switch to a different preferred numerosity. (d) Shows a similar reorganization plot for neurons that are non-selective numerosity neurons pre-training. (e) Shows a similar reorganization plot for neurons that were not numerosity sensitive pre-training. **L**, **M** Proportion of number-sensitive neurons in each layer and training epoch that are (a) P-SPONs, (b) New+Switch, and (c) neither (transient). The contribution of New+Switch increases as we move across higher layers and increases with training epoch. Source data are provided as a Source Data file.

We used a nDNN model, which had previously been pre-trained on visual object recognition^32^, and trained it to categorize non-symbolic representations of quantities. Images used in numerosity training consisted of a visual array of dots ranging from 1 to 9 with variable dot size, color, and location of dots (Fig. 1, see “Methods” section for details). The relationship between numerosity, and total area of dots, dot size, area of convex hull, and dot density respectively, were balanced across conditions, leveraging recent advances in algorithms for numerosity stimulus generation^33,34^. This ensured that no single perceptual feature was consistently correlated with numerosity across all conditions. We then assessed the accuracy of the nDNN in classifying previously unseen configurations of the non-symbolic dot stimuli for numerosities 1–9. Prior to numerosity training, numerosity classification rates were 13.1%, close to the chance level of 11%. Performance increased to 77.0% after the first training epoch. After 5 training epochs, performance on test examples reached 97.5%, eventually reaching an accuracy of 99.6% after 50 epochs (Fig. 3A).

These results demonstrate that with learning, performance of the model on non-symbolic to symbolic quantity mapping increased from chance levels prior to numerosity training to high levels of accuracy after just a few epochs of training.

### Reorganization of neurons post numerosity training

Our next goal was to determine how numerosity training alters numerosity tuning of SPONs in each of the four layers of the nDNN. Number-sensitive neurons were identified in two ways – first, those that showed a significant difference in activation across different input numerosities, but not across different stimulus conditions (selective numerosity neurons, identified via a 2-way ANOVA, see Methods for details, reorganization shown in Fig. 3A–E), and second, those that showed a significant difference in activation across different input numerosities regardless of whether they were also sensitive to stimulus conditions (all numerosity neurons, identified via a 1-way ANOVA, see “Methods” for details, reorganization shown in Fig. 3F–J). Note that the second classification subsumes the first.

We observed that the proportion of selective number-sensitive neurons decreased in higher layers V3 and IPS, compared to in V1 and V2 (Fig. 3B), whereas the number of all numerosity neurons increased in V3 and IPS compared to V1 and V2 (Fig. 3G). Training related increases in the number of selective number-sensitive neurons occurred primarily in V3 and IPS (Supplementary Table S1). Importantly, however, SPONs were not stable, but underwent significant reorganization with numerosity training, especially in layers V3 and IPS. We conducted several additional analyses to characterize the underlying changes.

SPONs that retain their preferred numerosity after training are referred to as P-SPONs. SPONs as well as P-SPONs are identified both based on *selective* as well as *all* numerosity neurons. First, we found that only 14.5% (V1), 17.2% (V2), 6.1% (V3), and 2.9% (IPS) of the selective SPONs retained their preferred numerosity after training (Fig. 3C and Supplementary Table S1). The remaining selective SPONs either dropped their selective number sensitivity (66% V1, 56% V2, 71% V3, 80% IPS), or switched their preferred numerosity (19% V1, 27% V2, 23% V3, 17% IPS). Next, we found that only 19.7% (V1), 33.5% (V2), 25.5% (V3), and 13.6% (IPS) of all SPONs retained their preferred numerosity after training (Fig. 3H and Supplementary Table S2). The remaining SPONs either dropped their selective number sensitivity (55% V1, 26% V2, 7% V3, 0% IPS), or switched their preferred numerosity (25% V1, 41% V2, 68% V3, 86% IPS).

Selective SPONs seem more likely to drop number sensitivity with training, whereas overall SPONs were more likely to switch numerosity with training. To further analyze this, we evaluated how neurons reorganized between selective and non-selective (jointly sensitive to both numbers and stimulus condition) numerosity across layers (Fig. 3K and Supplementary Table S3). Post training, the proportion of neurons that were neither selectively nor non-selectively numerosity sensitive reduced across layers (59% in V1, 26% in V2, 8% in V3, 0% in IPS). The proportion of neurons switching from non-numerosity to selectively numerosity sensitive neurons post-training also reduced across layers (14% V1, 8% V2, 2% V3, 0.5% IPS).

Even though training was based only on numerosity, with balanced stimuli across conditions, the proportion of non-selectively numerosity sensitive neurons remained high post training, especially in the higher layers (12% V1, 39% V2, 68% V3, 86% IPS). Switching between selective and non-selective numerosity neurons was relatively low (8% V1, 21% V2, 25% V2, 17% IPS), but switching between which numerosity a neuron was sensitive to, was higher (12% V1, 32% V2, 61% V3, 84% IPS).

We then examined neurons that were not spontaneously and selectively tuned to numerosity prior to training (non-SPONs), and found that 73% (V1), 69% (V2), 77% (V3), and 86% (IPS) retained a lack of selective numerosity preference (Fig. 3D). Examining the number sensitive neurons post-training (Fig. 3E, J), we found that the New+Switch neurons as a proportion of post-training number sensitive neurons was high across layers when measured for both selective (between 85% to 99%) and all (between 65% to 87%) numerosity neurons, with the remaining being P-SPONs.

Finally, confusion matrices based on the proportion of SPONs that were number-sensitive before and after training, to either the same or different preferred numerosities confirmed high levels of reconfiguration across all numerosities. This is demonstrated by the lack of strong diagonal elements (Supplementary Fig. S1).

These results demonstrate that training leads to significant reconfiguration of numerosity preference across all four layers of the network architecture, with distinct patterns of reorganization in the lower layers V1 and V2, versus higher layers V3 and IPS.

### Dynamics of training-related reorganization

Next, we examined how training-related reorganization evolves across epochs. We examined dynamic changes in three groups of neurons, (i) persistent SPONs (P-SPONs): SPONs that were number sensitive in the pre-trained network and continued to be number sensitive for the same preferred numerosity in the currently selected epoch; (ii) New+Switch: neurons that were number-sensitive for a particular numerosity during the current selected epoch, were not number-sensitive for this numerosity pre-training, and remained number-sensitive for the same numerosity at the end of training; and (iii) transient: neurons that were number sensitive at a given epoch but did not meet the criteria for being either P-SPONs or New+Switch.

We found that the proportion of P-SPONs dropped dramatically after epoch 0 in all layers and then stabilized across subsequent training epochs (Fig. 3L, M and Supplementary Fig. S2). The change from pre to post numerosity training was from 27% to 4% in V1, from 29% to 5% in V2, from 13% to 1% in V3, and from 6% to 0.2% in IPS, for selective P-SPONs. Interestingly, over 50% (Fig. 3L, right panel) of each layers’ selective number sensitive neurons were transient number sensitive, that is, neither P-SPONs, nor do they remain number sensitive for specific numerosities after training.

These results demonstrate that training involves continuous dynamic reorganization, and this process continues even when accuracy levels are roughly stable across epochs. This pattern of dynamic reorganization was especially higher in layers V3 and IPS, which showed a higher proportion of newly trained and switched neurons across training epochs.

### Ablation analysis to determine role of persistent spontaneous neurons in IPS after training

We conducted an ablation analysis by removing connections between layer IPS and the final output layer to determine the relative importance of P-SPONs, that is, neurons which were spontaneously number sensitive prior to number training and maintained their numerosity preference after training. Ablating only the P-SPONs resulted in no perceivable reduction of network performance (Fig. 3A, F, pink line), with saturation performance accuracy reaching 99.7%. On the other hand, ablating all neurons but keeping only the P-SPONs led to a significant drop in performance (Fig. 3A, F, green line) to an accuracy of only 25%.

These results demonstrate that P-SPONs in layer IPS are not critical for numerosity classification. and that their importance to the network gradually reduces with increased numerosity training.

### Changes in the precision, stability, and selectivity of number sensitive neurons with training

**Fig. 4:**
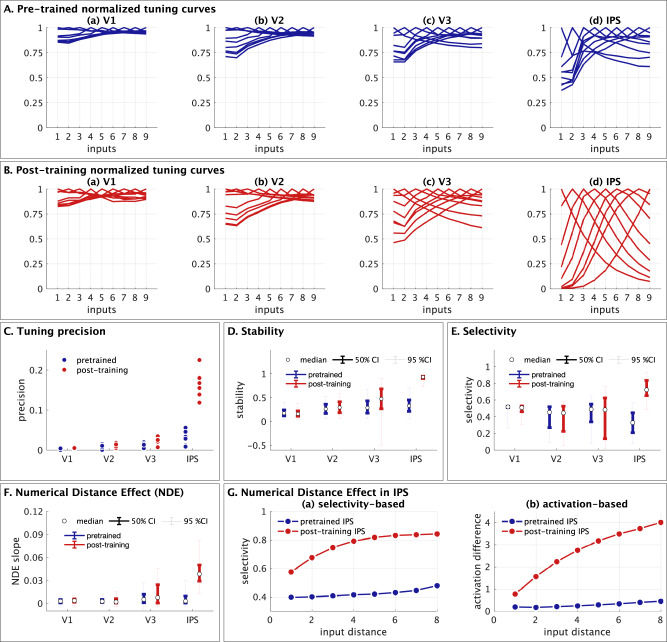
Changes in key neuronal properties of selective number sensitive neurons with numerosity training. **A** Normalized tuning curves for the pre-trained network: The plots show the mean normalized activation values (by input stimuli (1 to 9, on the *x*-axis), grouped by neurons of each preferred numerosity (PN), for layers (a) V1, (b) V2, (c) V3, and (d) IPS. **B** Normalized tuning curves for the post-training network for layers (a) V1, (b) V2, (c) V3, and (d) IPS. **C** Tuning precision: Acuity of the tuning curves as measured by the weighted average precision of the best fitting Gaussian tuning curves for each numerosity. Training leads to improving precision across layers, but primarily in IPS. Each dot represents the tuning precision for a preferred numerosity from 1 to 9. **D** Stability: the rank correlation (Kendall’s tau) or preservation of relative rank order of numerosities across conditions. Values >0 indicate better than chance level agreement, and values close to 1 indicate almost perfect rank order preservation across conditions. Improvements in stability increase as we move from V1 to IPS. **E** Selectivity: the proportion of comparisons where neuronal responses are higher for the PN. Higher selectivity indicates higher consistency of preference for the PN. Selectivity improves post training only in the IPS layer. **F** Numerical distance effect (NDE) is calculated as the average slope of selectivity versus input distance for each neuron, and averaged over number sensitive neurons. Improvements in NDE are highest in the IPS layer. The circles represent median values, the thick bars show the IQR (50% CI) and the thin bars show the 95% CI. **D**–**F** The circles represent median values, the thick bars show the IQR (50% CI) and the thin bars show the 95% CI. **G** NDE in the IPS layer: (a) Average selectivity and (b) Activation difference are both shown as a function of the numerical distance between pairwise input stimuli. The distance effects increase sharply from pre-trained (blue) to post-training (red). Source data are provided as a Source Data file.

Next, drawing on prior electrophysiological studies^35–37^, we examined several key properties of the individual selective number sensitive neurons and how they changed with training (Supplementary Table S4 and Fig. 4).

First, we examined tuning curves for number-sensitive neurons and investigated how tuning properties (see “Methods” section for details) in layers V1, V2, V3, and IPS are altered by numerosity training. We found that the neuronal tuning curves became sharper with numerosity training (Fig. 4A, B) and both their precision (acuity) and level of improvement in acuity post-training, increased progressively across layers (Fig. 4C), significantly so in the IPS layer.

Second, we measured the stability of number sensitive neurons based on their relative tolerance for how well they preserved responses across identity preserving transformation^35^. The measure used was Kendall’s tau (see “Methods” section for details), which measures rank order agreement across the eight conditions, with higher relative tolerance indicating that the differences in condition did not alter the relative numerosity order preference of neurons. The pre-trained network had a low level of variability in stability across layers, but after training, there was a significant increase in stability across the visual information processing hierarchy (Fig. 4D). A two-way ANOVA revealed an interaction between training epochs and nDNN layers (${F}_{{{{{\mathrm{3,200125}}}}}}$ = 7294, *p* < 0.0001), with a significant increase in differentiation between layers after training, as stability in V1, V2, and V3 showed minimal changes, while stability in layer IPS increased dramatically from 0.344 to 0.914.

Third, we measured the selectivity of number sensitive neurons by comparing pairwise activations of each neuron for different configurations of preferred and non-preferred numerosities and measuring the proportion of comparisons where the activation of preferred numerosity is higher (see “Methods” section for details). A neuron that always results in higher activation for any configuration of the preferred numerosity than for any other configuration of any other numerosity will have a maximum selectivity (of the preferred numerosity) of 1. The average selectivity across neurons only increases significantly with training in the IPS layer from 0.327 to 0.733 (Fig. 4E).

Supplementary Table S5 shows the same analysis but for all number sensitive neurons, and all key trends identified based on the selective number neurons are also replicated for all number sensitive neurons.

Together, these results demonstrate that precision, stability and selectivity of neurons increased with numerosity training, particularly in IPS. Clear differences along the information processing hierarchy from V1 to IPS emerged only after numerosity training. Further, neurons in IPS showed a wide range of values of precision, stability, and selectivity.

### Changes in numerical distance effect with training in layer IPS

Next, we examined the inferred numerical distance effect^38^ in layer IPS neurons by computing the selectivity (proportion of pairwise comparisons where preferred numerosities show higher activation) of each neuron as a function of the distance between preferred and non-preferred inputs being compared. The average slope of the selectivity versus input distance was computed as the numerical distance effect (NDE). NDE was low before training but increased after training, especially in the layer IPS (Fig. 4F) from 0.006 to 0.041. In layer IPS, post-training, both selectivity (Fig. 4Ga) and difference in neuronal activation (Fig. 4Gb) increased significantly as the distance between input pairs increased, although the selectivity saturated at high input distances. The average slopes in IPS layer were much higher post-training compared to pre-training.

Similarly, we measured the numerical size effect (Supplementary Fig. S4), separately for each distance value. The size effect computes the same measures but as a function of input size (sum of input pairs) instead of input distance, and shows a similar effect, with much stronger size effects in IPS post-training, compared to the pre-trained network.

These results demonstrate that numerical distance and size effects that are typically observed in humans and primates, emerged in the nDNN, and were primarily observed in the IPS layer post numerosity-training.

### Differences between selective and non-selective numerosity neurons in the IPS layer

While the key neuronal characteristics (tuning precision, stability, selectivity, and NDE) are similar for selective and all numerosity neurons, the relationship between these characteristics at a neuronal level is different. We measured the correlation between selectivity, tuning precision, stability, and NDE at a neuronal level pre and post training, separately for selectively number sensitive neurons, for non-selectively (or conjunctively) number sensitive neurons, and for all number sensitive neurons in the IPS layer (Supplementary Table S6 and Supplementary Fig. S3). The correlation between tuning precision and stability, as well as between tuning precision and NDE, reduces with training for selectively number sensitive neurons, but increases with training for the non-selective number sensitive neurons, and for all number sensitive neurons in the layer.

### Post-training tuning in nDNN layer IPS resembles numerosity tuning observed in monkey and human IPS

**Fig. 5:**
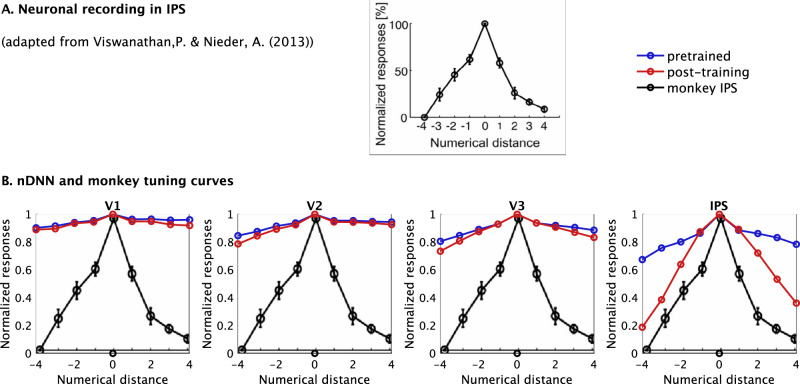
Correspondence between selective numerosity tuning curves in layer IPS of nDNN model and IPS subdivision of parietal cortex in monkeys. **A** Normalized tuning curve for numerosity in monkey IPS adapted from Viswanathan & Nieder^39^. All rights reserved. © PNAS 2013. Error bars indicate SEM. **B** Normalized tuning curves in our nDNN model (Fig. 4A, B) averaged across all numerosities showing tuning curves up to a distance of 4 units from the preferred numerosity, compared to tuning curves in monkeys. Numerosity tuning curves in layer IPS of the nDNN were similar to the those reported in the IPS of monkeys. Layer IPS units showed high-levels of similarity only after training. V1, V2, and V3 units did not show similarity with neuronal recordings either prior to after training. The normalized tuning curve (black) for numerosity in monkey IPS adapted from Viswanathan and Nieder^39^. All rights reserved. © PNAS 2013. Error bars indicate SEM. Source data are provided as a Source Data file.

**Fig. 6:**
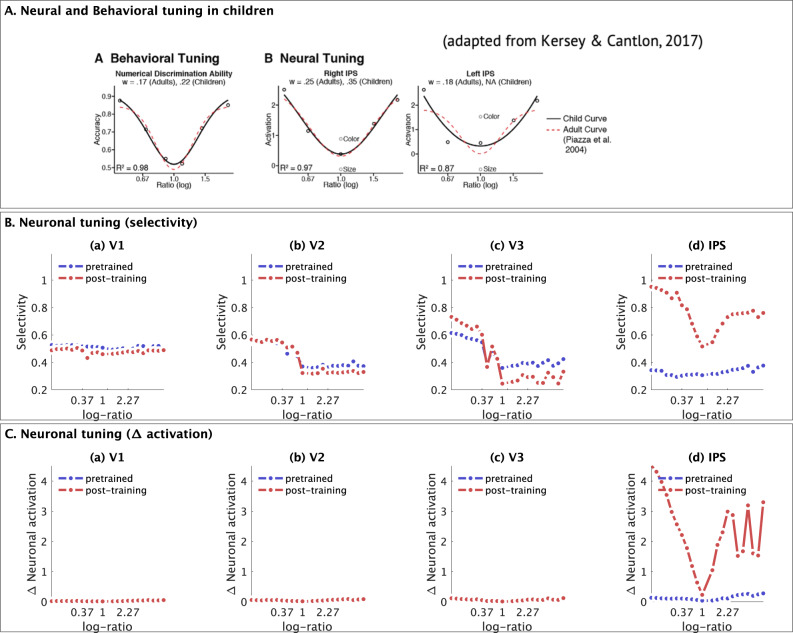
Neural tuning in nDNN as a function of *log*-ratio numerosity. **A** (A) Behavioral tuning for number discrimination and (B) inferred neural tuning in the IPS, as a function of the log-ratio of input numerosities, for adults and children. Tuning curves demonstrate a U-shaped curve with slightly higher tapering (steeper rise away from $\log$-ratio 1) for adults compared to children. Adapted from Kersey & Cantlon^40^. **B** Neuronal tuning based on selectivity as a function of $\log$-ratio of input numerosities being compared shows a similar sharp U-curve in the layer (d) IPS of our nDNN model, but not in the earlier layers (a) V1, (b) V2, and (c) V3 (blue: pre-trained; red: post-training). **C** Neuronal tuning based on difference in activation as a function of $\log$-ratio of input numerosities being compared shows a similar sharp U-shaped curve in layer (d) IPS of our nDNN model but not in the earlier layers (a) V1, (b) V2, and (c) V3 (blue: pre-trained; red: post-training). Source data are provided as a Source Data file.

Next, we sought to determine whether tuning properties of neurons in each nDNN layer resembled numerosity tuning curves observed in the monkey IPS regions^39^. We found highly tuned curves in layer IPS after, but not before, numerosity training (Fig. 5). This overlap was specific to layer IPS as no such tuning was observed in layers V1, V2, and V3. Furthermore, the tuning properties in layer IPS are also similar to those observed in behavioral and functional brain imaging studies in children and adults (Fig. 6A)^40^. Specifically, tuning based on selectivity (Fig. 6B), which effectively measures how often neurons show a stronger activation in response to their preferred numerosities, and that based on the actual difference in neuronal activation in response to preferred versus non-preferred numerosities (Fig. 6C), both as a function of the $\log$-ratio of each pair of input numerosities being compared, showed similar U-curves in neuronal tuning within the nDNN IPS layer, as the U-curves observed in behavioral tuning and neural tuning with the IPS of humans. Similar patterns were not however observed for the pre-trained and post-training tuning curves in the nDNN V1, V2, and V3 layers, or in the pre-training curves in the IPS.

These results demonstrate that neuronal tuning in layer IPS layer post-training resembles neuronal properties observed in the IPS in both humans and non-human primates.

### Neural representational similarity between numerical quantities

**Fig. 7:**
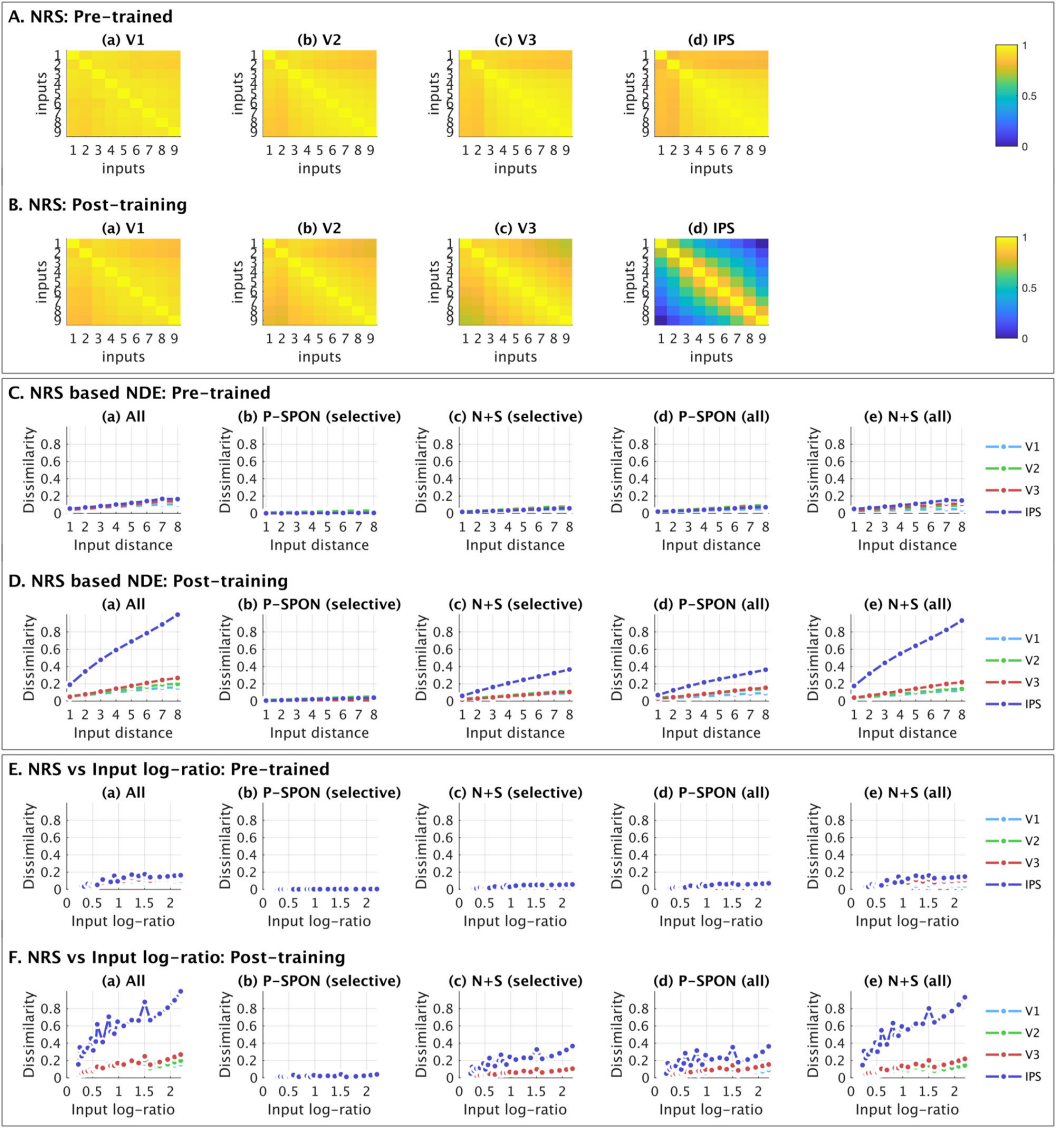
Neural Representation Similarity of distributed population-level response and relation to Numerical Distance Effect. **A**, **B** Neural representation similarity (NRS) calculated based on pairwise similarity between the mean activation across neurons in each layer (a) V1, (b) V2, (c) V3, and (d) IPS, for each value of the input stimuli. This shows us how well differentiated each input stimuli are, compared to other input values, in terms of their neuronal representations, pre-training (**A**) and post-training (**B**). The influence of training in the whole layer level RSA can be seen strongly in IPS, to a progressively smaller extent in V3 and negligible in V2 and V1. **C**, **D** The NRS is condensed to map the dissimilarity (1- average NRS) averaged as a function of each unique value of difference between inputs, that is, directly measure the numerical distance effect between representations of numerosities at a distributed level. A robust representation should show a sharply increasing linear trend in the average similarity with increasing input difference. The condensed RSA as a function of input difference is calculated for (a) the whole layer, (b) P-SPONs based on selective numerosity neurons, (c) New+Switch based on selective numerosity neurons, (d) P-SPONs based on all numerosity neurons, and (e) New+Switch based on all numerosity neurons. Pre-training (**C**), these linear trends have a very small slope. This slope increases with numerosity training (**D**), with significantly larger increases as we move from lower to higher layers, especially in IPS. **E**, **F** The NRS is condensed to map the dissimilarity (1- average NRS) averaged as a function of each unique value of $\log$-ratio between inputs, that is, directly measure the ratio effect between representations of numerosities at a distributed level. This is shown for (a) the whole layer, (b) P-SPONs based on selective numerosity neurons, (c) New+Switch based on selective numerosity neurons, (d) P-SPONs based on all numerosity neurons, and (e) New+Switch based on all numerosity neurons. A robust representation should show a sharply increasing linear trend in the average similarity with increasing $\log$-ratio. Source data are provided as a Source Data file.

Our third major goal was to go beyond coding at an individual neuron level, and examine the distributed coding properties across all neurons in each layer. To do this, we conducted representational similarity analysis (RSA) of responses across all neurons in each layer, regardless of their individual tuning properties, similar to the approach used in neuroimaging studies (see Methods for details). Prior to numerosity training, there was negligible differentiation in any of the layers (Fig. 7A). After numerosity training (Fig. 7B), layer IPS showed higher differentiation in the neural representations of inputs, characterized by a diagonal pattern of neural representational similarity and weak overlap across numbers. In contrast, layers V1, V2 and V3 showed very little differentiation after training. A similar RSA pattern was observed in the subset of New+Switch neurons, but the differentiation post-training in the IPS layer was much more diluted in the subset of P-SPONs (Supplementary Figs. S5 and S6).

We then investigated the average RSA based dissimilarity between representations for each unique value of distance between inputs (Fig. 7C, D), and $\log$-ratio between inputs (Fig. 7E, F). The average slope of this representational dissimilarity as a function of input distance ($\log$-ratio) is similar to the NDE measure, but rather than calculating it at a neuronal level, this provides a measure of the NDE (ratio-effect) based on population level distributed encoding. We found increased distributed NDE and $\log$-ratio effect post numerosity training primarily in IPS (Supplementary Table S7), with significantly larger increases based on New+Switch compared to P-SPON neuronal groups (Fig. 7D). Importantly, comparing the NDE at a neuronal level and distributed level shows that distributed NDE in layer IPS post numerosity training was more than double NDE at the single unit level (0.041 neuronal NDE versus 0.108 distributed NDE), showing that the distributed representations provide additional discriminative power over the neuronal representations.

The NDE and $\log$-ratio effects were also stronger for all numerosity neurons compared to only selective numerosity neurons (Fig. 7De, Fe versus 7Dc, 7Fc).

These results demonstrate the importance of newly trained and numerosity-switching neurons, rather than P-SPONs, to differentiating between numbers at a population level, and also highlight the importance of all number neurons including those that are not selectively sensitive to numerosity. Results further reveal that training changes distributed representations primarily in layer IPS, and that distributed population level encoding increases NDE beyond neuronal measures.

### Latent structure of distributed neural representations

Our fourth goal was to determine the latent structure of distributed neural representations. We used multidimensional scaling (MDS) which allowed us to uncover low-dimensional representations underlying numerosity. Specifically, MDS allowed us to reduce the original dimensionality from approximately 200k (V1), 100k (V2), 50k (V3), and 25k (IPS), reflecting the number of neurons in each layer, to a very small number of dimensions. MDS eigenvalues and derived goodness of fit^41^ revealed that 2 dimensions provide robust representations (*g* ranges from 0.70 to 0.97 across layers for 2 dimensions; see Methods, Supplementary Table S8). Thus, MDS reduced the average representation of each input stimuli (1–9) from the number of neurons in each layer to a latent two-dimensional space.

**Fig. 8:**
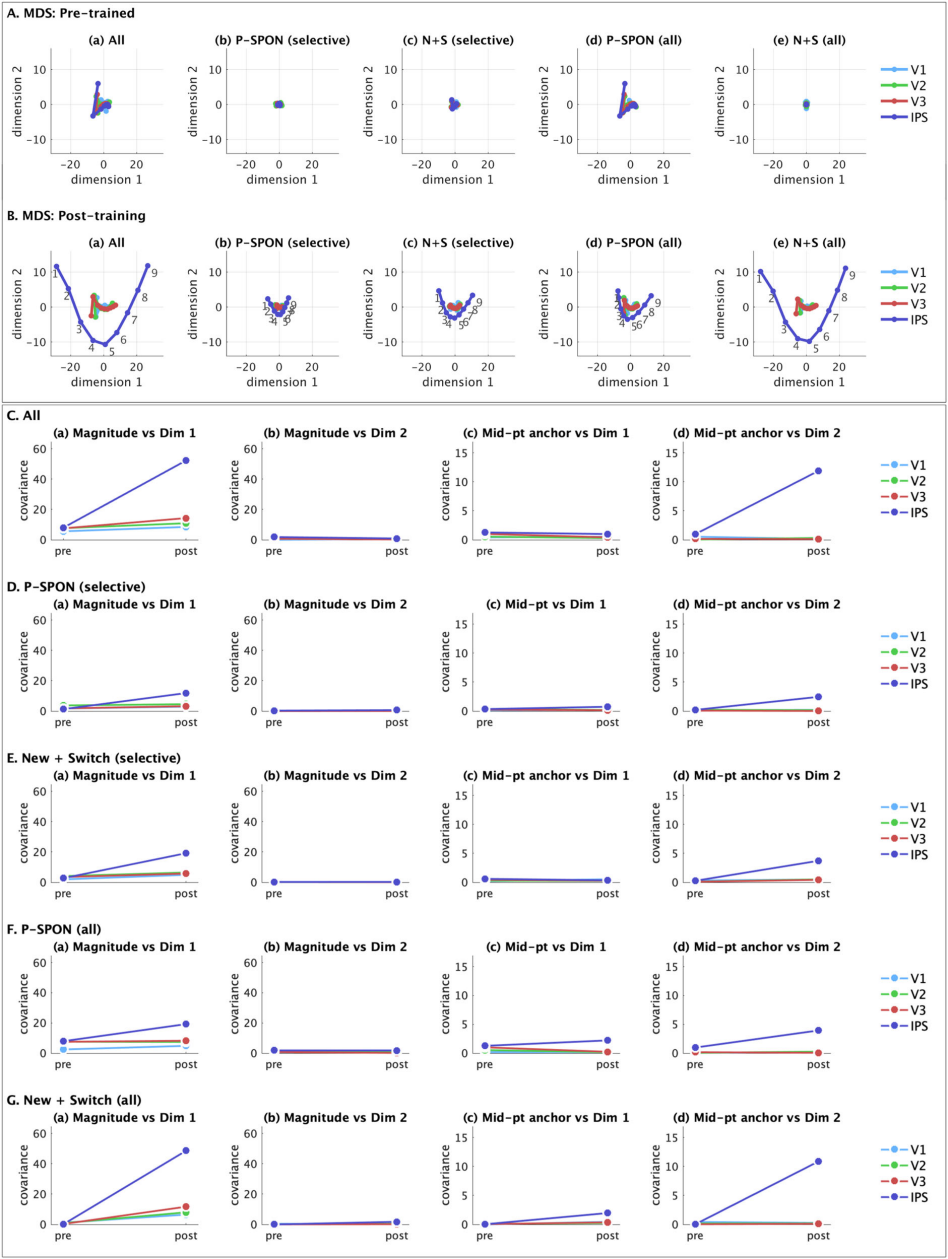
Multidimensional scaling of population-level responses reveals latent two-dimensional representations of absolute and relative magnitude. **A**, **B** Multidimensional scaling (MDS) reveals a low-dimensional representation of each input stimuli (1–9) in a two-dimensional space. The two-dimensional representations of each input at each epoch were obtained from multiple groups of neurons, using (a) the whole layer, (b) P-SPONs based on selective numerosity neurons, (c) New+Switch based on selective numerosity neurons, (d) P-SPONs based on all numerosity neurons, and (e) New+Switch based on all numerosity neurons, for **A** pre-trained and **B** post-training networks. The two-dimensional representations are color coded by layer (V1: light blue, V2: green, V3: red, IPS: dark blue). The MDS reveals the emergence of a clear two-dimensional arch structure in V3 and especially in IPS, post numerosity training. This structure is not present for the pre-trained network. This representation also shows that whilst whole layer representations depend on SPONs pre-training (compare Aa and Ad), they shift to newly trained and switched neurons post training (compare Ba and Be), as well as the fact that they depend not just on the selective numerosity neurons (b, c) but on all numerosity neurons including those sensitive to both numerosity and condition (d, e). **C**–**G** The two dimensions obtained from the MDS using (**C**) all neurons, (**D**) P-SPONs based on selective numerosity neurons, (**E**) New+Switch based on selective numerosity neurons, (**F**) P-SPONs based on all numerosity neurons, and (**G**) New+Switch based on all numerosity neurons, are evaluated for covariance with key input stimulus properties. These include the stimulus magnitude (perceptual property), and distance of the input from the mid-point of the stimuli space (cognitive property). Across these sets the pre-training covariances are low, but post-training covariance between (a) dimension 1 and magnitude, and between (d) dimension 2 and distance from mid-point of the stimuli space, increase post-training in the IPS layer, especially in (A) and (G). The covariance between (b) dimension 2 and magnitude, and (c) dimension 1 and distance from mid-point remain low post-training. Source data are provided as a Source Data file.

MDS revealed that prior to numerosity training, all layers showed minimal differentiation between numbers (Fig. 8A). After training, a clear two-dimensional structure emerged in layer IPS. Low dimensional representations were less differentiated in layers V1, V2, and V3. Analysis of different sub-populations of neurons revealed that effects were driven by New+Switch number-sensitive neurons (Fig. 8B), rather than P-SPONs.

Together, these results demonstrate that numerosity training results in the emergence of a two-dimensional latent representational structure in layer IPS.

### Coding properties of the latent two-dimensional representations

A closer examination of the latent structure of representations obtained via MDS revealed two distinct codes for absolute and relative magnitude. We examined the covariance (Fig. 8C–G) between each latent dimension, and two key properties of numerosity coding: magnitude (1–9), and distance from mid-point of the stimuli set. The first is an absolute measure of the non-symbolic to symbolic mapping, while the second is a relative measure characterizing the structure of the stimuli set with a salient reference or anchor point^42^. Specifically, the relative magnitude refers to a representation of the structure of the stimulus space with respect to the mid-point, and a representation of numbers within this space based on distance from this anchor.

The first latent dimension (Dimension 1) showed a high covariance with the absolute magnitude of numerosity, and this increased significantly with numerosity training. Dimension 1 did not show strong covariance with the distance of numerosities from the mid-point anchor in any layer. The second latent dimension (Dimension 2) showed a strong covariance with distance from the mid-point anchor, but not the absolute magnitude. These effects were observed only after training and in layer IPS. Further, while new and switched neurons showed similar effects to the whole layer, the covariance patterns for P-SPONs were similar but relatively muted.

**Fig. 9:**
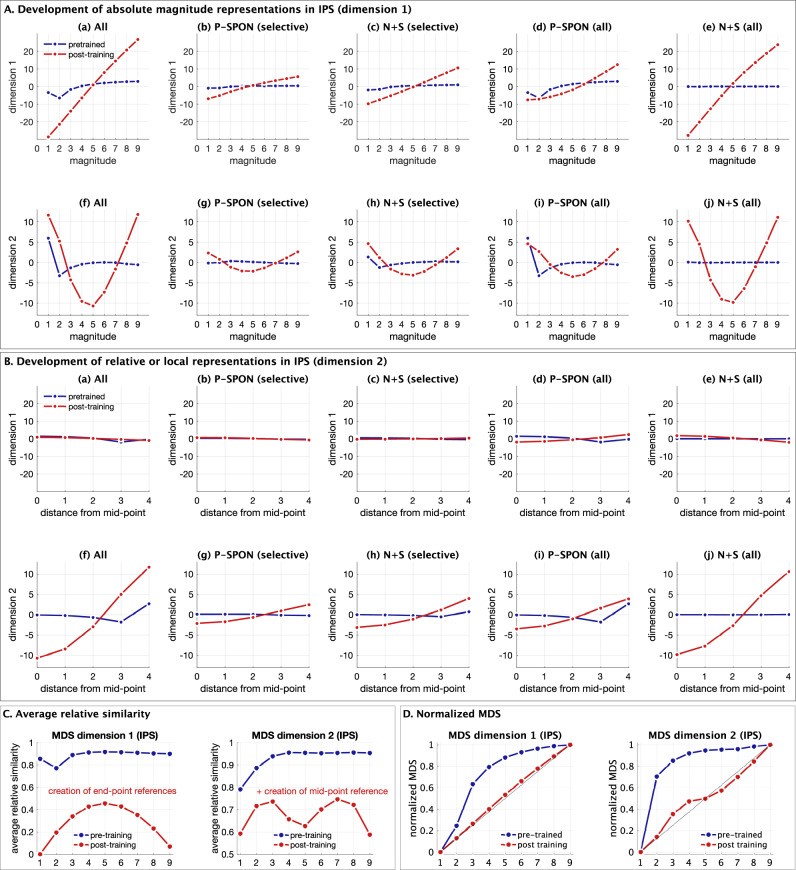
Multidimensional scaling of population-level responses reveals magnitude and midpoint anchoring in layer IPS of the nDNN. **A** The plots show each MDS dimension as a function of the input stimuli magnitude, pre (blue) and post (red) training. The first dimension encodes a unimodal monotonic representation of the input stimuli magnitude. **B** The plots show each MDS dimension as a function of the distance from mid-point of the stimuli space, pre (blue) and post (red) training. The second-dimension codes for the distance of the input from the mid-point, with the response profile showing that representation increases faster as the distance from mid-point increases. For both (a) and (b), the dimensions are shown for (a, f) the whole layer, (b, g) P-SPONs based on selective numerosity neurons, (c, h) New+Switch based on selective numerosity neurons, (d, i) P-SPONs based on all numerosity neurons, and (e, j) New+Switch based on all numerosity neurons, with a–e showing dimension 1, and f–j showing dimension 2. **C** For each of the two MDS representational dimensions in layer IPS, the distance between each pair of input values is calculated, and the average distance of each input from all other values is converted into a relative similarity measure. High relative similarity of an input value implies higher propensity to confuse the input with other input values, and thus influence the output variability and errors. Training reduces the similarity between inputs, but also changes the shape of the similarity curves. It reduces the average similarity of the end-points (dimension 1), and reduces the similarity of the mid-point of the stimuli space (dimension 2). **D** The MDS dimensions for each numerosity can be translated to measure the “distance” between consecutive numerosities and create a latent “number line” in each MDS dimension. This is normalized and shown in the plots, for the pre-trained and post-training MDS representations. The pre-trained number lines show a logarithmic shape. The post-training number lines have a near-linear profile in the first dimension, and cyclic profile with a mid-point anchor (reference point) in the second dimension. Source data are provided as a Source Data file.

We further probed the two latent dimensions of numerosity in layer IPS by measuring responses as a function of numerical magnitude (Fig. 9A), and distance from mid-point of the stimuli space (Fig. 9B). This analysis revealed that representations along Dimension 1 increased linearly with magnitude, but not with distance from the mid-point. In contrast, representations along Dimension 2 increased monotonically with distance from mid-point, but not with magnitude. Changes in both dimensions were driven by New+Switch neurons rather than P-SPONs. We performed additional control analysis to further validate our finding of mid-point anchoring associated with MDS Dimension 2. We computed distance with respect to all potential anchors ranging from 1 to 9. Only the use of the mid-point 5 as an anchor resulted in a linear monotonic increase in this dimensions that is symmetrical for increasing distances in both directions. Using other values (1–4 or 6–9) as anchors does not produce this profile (Supplementary Fig. S7). Supplementary analysis also shows that recursion within the network amplifies the MDS representations and resulting covariance patterns of the latent dimensions (Supplementary Fig. S8).

Together, these results provide further evidence for distinct coding mechanisms for magnitude and distance, and highlight the emergence of an anchor at 5, the midpoint of the training input set.

### Emergence of a latent number line representation in layer IPS of the nDNN

Next, for each MDS dimension, we examined the similarity of representations across numbers. We calculated the similarity of MDS representations of each number (1–9) with all other numerosities. High relative similarity of a number on a particular dimension implies higher propensity to confuse the number with other values, and thus has the ability to influence the variability and errors in response to numerical values. Our analysis revealed three key findings (Fig. 9C). First, similarities along both Dimensions 1 and 2 reduced with training, thus increasing the precision with which each input can be identified. Second, the “flatness” in MDS Dimension 1 similarity observed prior to training was eliminated after training, and is characterized by the formation of an inverted U-shape. Third, similarity in MDS Dimension 2 was characterized by an inverted “W” shape, with the end points and mid-point showing lower similarity, thus establishing three reference points (1, 5, and 9) that are more differentiated.

The MDS dimensions can serve as a proxy for a latent “number line” emerging in the nDNN, based on distance between consecutive numbers in the MDS space. This is normalized and plotted in Fig. 9D, and shows that for pre-trained neurons, the latent number line mapped on both MDS dimensions is a logarithmic or non-cyclic power law shaped function^31,42^, but post-training, this changes to close to linear on the first MDS dimension, and to a one-cycle function (i.e. with a middle reference point^31,42^) on the second MDS dimension.

These results demonstrate that numerosity training creates a latent number line representation in layer IPS, with a shift from logarithmic or power law shaped to single-reference point cyclic, and linear number line structures with learning, similar to that observed during human development.

### Latent distributed representations show lower representational drift over learning

Neuronal representational drift^43,44^ was measured as one minus the rank correlation coefficient (numerosity order preservation) across consecutive epochs for each neuron (see “Methods” section for details). A similar drift was measured at a distributed representation level by measuring one minus the coefficient based on the first two latent MDS dimensions for each layer. These measures were averaged across the first 10 training epochs (Supplementary Fig. S9) and show a reduction in drift across the visual processing hierarchy from V1 to IPS at the neuronal level (0.35 V1, 0.31 V2, 0.23 V3, 0.10 IPS). Importantly, the average representational drift measured at a distributed latent MDS level (0.20 V1, 0.01 V2, 0.03 V3, 0.02 IPS) is much lower than that measured at the neuronal level. A two-way ANOVA reveals a significant main effect of level of representation - neuronal versus latent MDS (${F}_{{{{{\mathrm{1,7}}}}}}$ = 17.2, *p* = 0.0255).

These results demonstrate that while changes in neuronal characteristics with numerosity training are stronger in the higher (V3 and IPS) layers, the neuronal representational drift is higher in the earlier layers (V1 and V2). Further, the representational drift in population level encoding is much lower than that at the neuronal level.

### Relationship between classification accuracy and network dynamics

Our fifth goal was to determine the link between dynamic changes in neural representations of the network during training and its behavioral performance. Network accuracy during numerosity training increased from 77% (after epoch 1) to 98.9% at epoch 10, after which it reached an asymptote, slowly reaching 99.7% by epoch 50. To capture neural and population-level features associated with significant shifts in accuracy, we analyzed the first 10 epochs and measured the relationship between network accuracy and network features, including the weighted average tuning precision of tuning curves, proportion of selective number sensitive neurons as a percentage of total layer neurons, the proportion of selective New+Switch neurons as a percentage of number sensitive neurons, and the average inter-stimuli representational distance along the first two dimensions of the MDS representation. These features are calculated for each of the four layers V1, V2, V3, and IPS, across these 10 epochs. Accuracy was correlated only with characteristics of layer IPS. Specifically, accuracy was positively correlated with MDS distance between numerosities (*r* = 0.81, *p* = 0.0042), and precision of the fitted Gaussian tuning curves for number sensitive neurons (*r* = 0.83, *p* = 0.0027), of the IPS layer. Accuracy was also related to the proportion of New+Switch number sensitive neurons (*r* = 0.80, *p* = 0.0052) when including all number sensitive neurons, regardless of whether they were also sensitive to stimulus condition, but accuracy was not correlated with the similar New + Switch proportion of purely selective number sensitive neurons.

These results demonstrate the importance of the IPS layer, the role of New+Switch neurons (as opposed to P-SPONs) and the relevance of all numerosity neurons (as opposed to selective numerosity neurons) in improving network performance.

### Control analyses

**Fig. 10:**
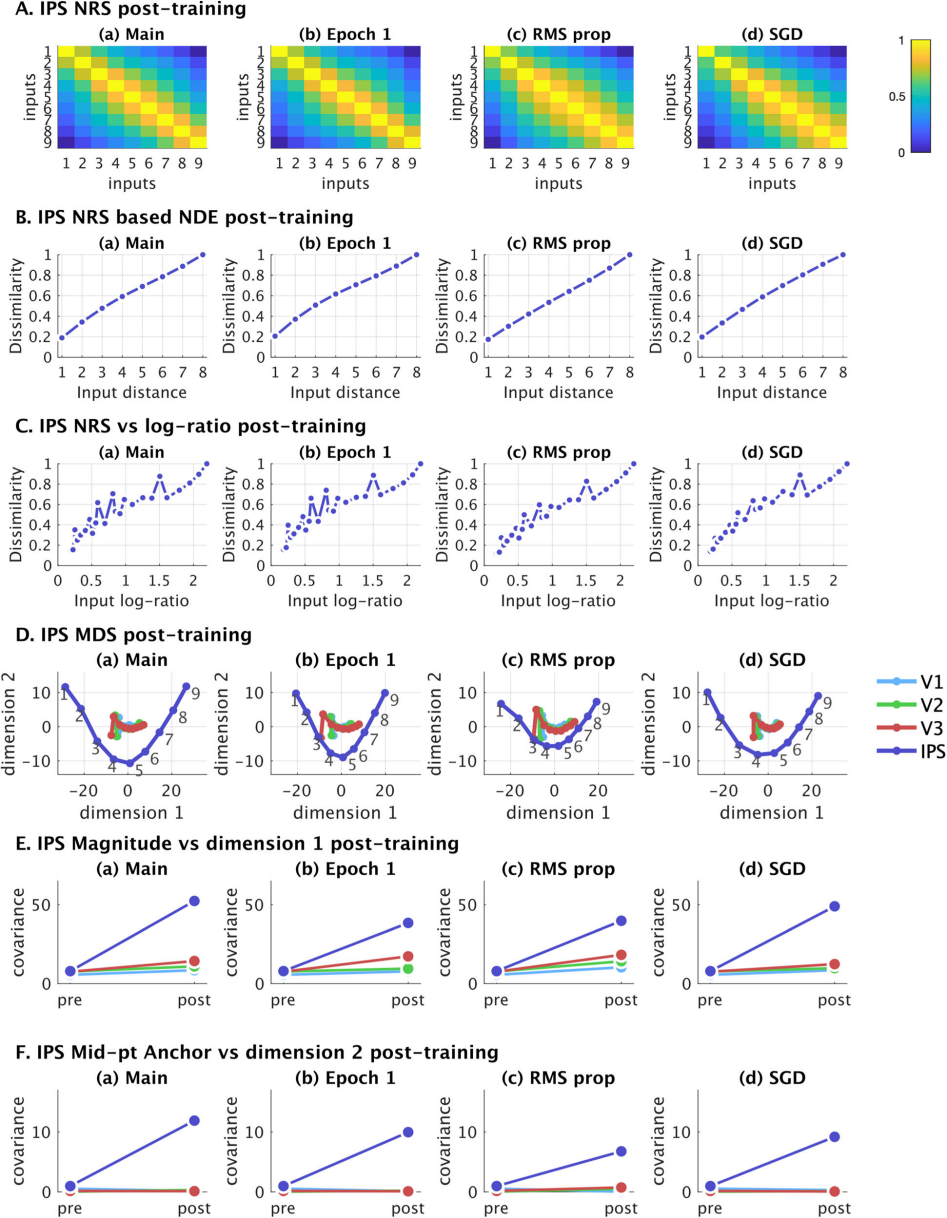
Control Analyses. The key distributed properties including **A** NRS representation, **B** NRS based distance effect, **C** NRS based $\log$-ratio effect, **D** MDS representations, **E** covariance between MDS dimension 1 and magnitude, and **F** covariance between MDS dimension 2 and mid-point anchor. These properties are all replicated for the three control analyses conducted, and in **A**–**F** the subplots show (a) Main: primary analysis based on accuracy reaching 99%+ levels; (b) Epoch 1: control for limited single epoch training where accuracy increases to 77%, similar to human accuracy levels; (c) RMS prop; control for change in training method from Adam optimizer to RMS propagation; and (d) SGD: control for change in training method from Adam optimizer to stochastic gradient descent. Source data are provided as a Source Data file.

We conducted three control analyses, measuring key aspects of reorganization, neuronal properties, and distributed population level properties to evaluate the robustness of our findings. First, since the 50 epochs of training results in high accuracies (over 99%), we conduct the analyses after just 1 epoch of training, where the resulting accuracy at 77% is closer to human levels of accuracy^45,46^. Secondly, to evaluate whether the specifics of the nDNN training algorithm affect these measures, we implement the same models with two variants of the learning algorithm (RMS propagation and stochastic gradient descent). The control analyses are detailed in the supplementary information (Supplementary Tables S9 and S10) and show that the key results relating to tuning properties of neurons and neuronal reorganization hold in all four control cases. Figure 10 compares the distributed representations and properties of these control analyses and shows that the NRS and MDS representations, and their derived properties remain stable across the control analyses.

## Discussion

We used a neurobiologically inspired nDNN architecture with hierarchically organized layers V1, V2, V3, and IPS, and recurrent connections (Fig. 1), to investigate how tuning of individual neurons and distributed population responses are reorganized by learning. We trained the nDNN to learn mappings between non-symbolic and symbolic representation of quantity, allowing us to probe how spontaneously tuned neurons change with numerosity training across the visual information processing hierarchy. Our magnitude-symbol mapping task constitutes a key advance over previous modeling work in this area. Our analysis revealed that training-related increase in performance was accompanied by extensive reorganization of neuronal response characteristics both at the level of individual neurons and at the population level, with increasing levels of network reorganization from layer V1 to IPS. Multidimensional scaling (MDS) of population level responses revealed two distinct low-dimensional representations that capture absolute and relative numerical magnitude, formation of reference points such as mid-point anchoring in numerical estimation, and emergence of changing number line representations^31,42^. Dynamic learning-related changes in neuronal tuning and distributed representations predicted accuracy of mapping between non-symbolic and symbolic numerosity, further highlighting how network reorganization contributes to learning. Our findings elucidate mechanisms by which learning builds novel representations supporting number sense.

The first goal of our study was to investigate how numerosity training changes neural representation of quantity. We showed that numerosity training resulted in significant reorganization and reduced the importance of spontaneous number-sensitive neurons. Previous studies have shown that monotonically coded numerosity information can emerge spontaneously from statistical properties of images in networks trained only on object recognition and in randomly initialized networks^6–8,29^. Crucially though, prior studies have not examined the emergence of number sense utilizing a domain-specific developmental paradigm similar to what children typically experience in which they learn mappings between non-symbolic and symbolic representations of quantity^15^. In a recent fMRI study, Schwartz and colleagues showed that neural representational similarity between symbolic and non-symbolic quantities predicted arithmetic skills in childhood^2^. Specifically, neural representational similarity in distributed brain regions, including parietal and frontal cortices and the hippocampus, was positively correlated with arithmetic skills. Thus, learning to map cross-format representations is a crucial developmental process. We trained the network to learn such mappings between non-symbolic and symbolic representations of quantity. This allowed us to explicitly measure the influence that domain-specific training, typical during development, has on neuronal representations of numerosity, and the role of spontaneous number sensitive neurons in developing cognitively meaningful representations that go beyond statistical features learned from early domain-general or naturalistic visual image processing.

We found that performance levels, which were at chance levels (~13%) prior to numerosity training, increased to accuracy levels over 99% within 50 epochs of training. Although the pre-trained network contains number sensitive neurons, it lacks the capability to correctly map non-symbolic inputs to symbolic numbers, without explicit training. This underlies the need for explicit learning, similar to developmental studies where the relationship between representations of symbolic and non-symbolic evolves over developmental stages and associated numerosity learning^2^. Our analysis uncovers how explicit learning influences the underlying representations of quantity at both the neuronal and population levels.

In an advance over previous studies, our training paradigm was implemented in a biologically inspired architecture which allowed us to probe training-induced changes in the properties of number sensitive neurons across V1, V2, V3, and IPS, along the dorsal visual processing hierarchy. Similar to previous studies^7^, our analysis revealed that the pre-trained nDNN also contained spontaneous number neurons (SPONs) based only on object recognition prior to any non-numerosity training. SPONs were found across all four layers, and purely number selective SPONs decreased across layers from 27% in V1 to 6% in IPS, whereas any number selective SPONs, regardless of whether they were also sensitive to other conditions, increased from 47% in layer V1 to 97% in IPS (Fig. 3B, G). Extending previous work, we found that domain-specific numerosity training results in significant changes to neuronal and distributed properties of the network, with massive reorganization of neurons, especially in the IPS layer.

We then examined the degree to which numerosity training altered the response properties of these SPONs in each layer. Crucially, after training, we observed a significant reorganization of neuronal response properties across all four layers characterized by the following features. First, only 3–17% of selectively number sensitive SPONs (14–33% of all SPONs) across each layer were tuned to the same numerosity after training (P-SPONs). Second, after training, P-SPONs constituted only 23% of selectively number sensitive neurons in layer V1 and 13% in IPS (14% and 1% based on all number sensitive neurons). Third, the early layers V1 and V2 displayed a large proportion of SPONs that were no longer tuned to any numerosity, as well as new number-sensitive neurons, after training. Fourth, layers V3 and IPS demonstrated a high proportion of neurons which switched preferred numerosities (Fig. 3C, H, K). Thus, the prevalence of SPONs that retained their numerosity preference after training was low across layers, and this proportion was lowest in layer IPS. Finally, ablation analysis revealed P-SPONs were irrelevant for network performance after training, while ablation of all neurons other than P-SPONs resulted in significant reduction of performance (Fig. 3A).

Together, these results suggest that SPONs are easily disrupted by even minimal training and that training reconfigures the numerosity related response properties of neurons in all network layers. Results also provide evidence for a fast network reorganization in parallel with development of increasingly high levels of accuracy in categorization of non-symbolic quantity. More generally, our findings suggest that spontaneous neurons arising from non-numerosity related visual experiences do not form an essential foundation for numerical cognition.

The second goal of our study was to investigate precisely how learning changes the tuning of neurons that code for numerosity. We showed that numerosity training increased acuity and stability of neuronal tuning. Our analysis revealed multiple features of learning dynamics. Notably, training resulted in improved neuronal tuning acuity primarily and most significantly in layer IPS. Studies of numerosity encoding in primate electrophysiological recordings have shown an approximate reduction of firing rate to 25% of peak firing for numerosities at a distance of 3 (Fig. 5A) from the preferred numerosity^4,39^. Numerosity neurons in layer IPS of our trained model showed comparable tuning curves (Fig. 5B). In contrast, in all other layers, tuning curves were flatter and barely dropped to below 50% that of the preferred numerosity (Fig. 4A, B).

Prior to training, nDNN tuning curves in the IPS layer showed low precision, averaging a standard deviation of 9.1 across numerosities, which is similar to that observed with AlexNet^8^ for numerosities 1–9. Post-training, the acuity of tuning curves in the IPS improved significantly, with a standard deviation of 2.47, which is similar to the range observed in the parietal cortex of monkeys for the numerosities 1–10^47^. Acuity of the tuning curves in the nDNN IPS layer post numerosity training thus improved, bridging the discrepancy observed between AlexNet neurons and numerosity representations in monkey neurons. Notably, neural tuning curves in layer IPS of the nDNN match the tuning curves observed in the IPS of monkeys^39^ (Fig. 5). These results were specific to layer IPS of the nDNN but not layers V1 to V3 (Fig. 6), highlighting a close correspondence between our biologically-inspired model and neuronal recordings.

We measured neuronal stability by assessing the relative tolerance of neurons to identity preserving transformations, including total dot area, convex hull area, and dot size. The relative tolerance measures how well each neuron maintains rank order preference for different numerosities, even when orthogonal perceptual properties of the numerosity are manipulated. This analysis builds on findings from neurophysiological studies demonstrating that “each neuron’s rank-order object preference under identity-preserving image transformations” is crucial for recognition, and that this response property is typically observed in neurons in higher visual areas, but not in early visual areas such as V1^36,48^. Using a similar metric, we found that training increased stability from 0.34 to 0.91 in nDNN layer IPS. In contrast, prior to numerosity training, stability was low in all layers with values ranging from 0.19 to 0.34 (Fig. 4D). Thus, training increases stability and generalizability, specifically in layer IPS across manipulations of dot area, convex hull area, and dot size. Additionally, layer IPS layer showed the lowest representational drift with learning (Supplementary Fig. S9), a finding consistent with electrophysiological recordings in monkey IPS^49^.

Together, these results demonstrate that numerosity training increases acuity and stability of neuronal tuning, in a manner consistent with neurophysiological observations, with a unique role for layer IPS post-training.

The third goal of our study was to go beyond coding properties of individual number sensitive neurons and determine how numerosity training alters distributed neural representations across neurons in each layer. We used representational similarity analysis (RSA) to capture similarity between patterns of neuronal activation for numerical stimuli taken pairwise. RSA revealed broadly tuned and overlapping representations between numerosity pairs (e.g., 2 vs 6 dots) in all four layers prior to training. With training, distinct representations emerged in layer IPS with low pairwise correlations between numerosity pairs (Fig. 7B). Together, these results demonstrate sharpening of neural dissimilarity between distinct numerical inputs.

To further characterize learning-related changes in population-level representations, we examined the numerical distance effect and the ratio effect at a distributed population level. Our analysis revealed a linear increase in representational dissimilarity with increasing numerical distance (Fig. 7C, D), and approximately linear increase with increasing $\log$-ratio of numbers (Fig. 7E, F), consistent with the emergence of robust number differentiation. The slopes of these linear profile were low prior to training, reflecting weak differentiation between numbers. The slopes increased progressively from V1 to IPS with training, and post-training differentiation was most prominent in layer IPS. Further analysis of neuronal subtypes revealed that improved differentiation in layer IPS representations were strongly driven by New+Switch neurons. Together, these profiles mimic effects consistently observed in behavioral studies, where the accuracy of non-symbolic number comparison increases, and reaction time decreases, as the distance between them (distance effect) or the ratio between them (ratio effect) increases. Further, the numerical distance effect observed at a distributed population level was higher than that observed at a neuronal level based on change in selectivity with input distance. Our findings are consistent with reports of distributed neural representations of visual numerosity in the IPS independent of the precise location of their receptive fields^50^.

Our findings suggest that behavioral distance and ratio effects are most strongly associated with plasticity of population-level neural activity in layer IPS and highlight the distributed and emergent nature of numerosity representations.

The fourth goal of our study was to investigate the latent structure of population level encoding of numerosity. To accomplish this, we used MDS to capture a low-dimensional representation of each layer for each of the input stimuli. MDS models the representational similarities between pairs of stimuli, such that each stimulus is a point in an n-dimensional space, with the distance in this n-dimensional space representing representational similarity between stimuli. Here, *n* is the number of neurons in each layer. MDS has been extensively used in psychology to represent cognitive maps and relations between stimuli and categories^51–56^.

MDS revealed a clear two-dimensional latent low-dimensional “horseshoe” representation in layer IPS that after training (Fig. 8B). Remarkably, the two-dimensional latent structure identified by MDS consisted of numerosity magnitude on one dimension, and distance from an anchor – the mid-point of stimulus space – on the second dimension (Figs. 8C and 9B). These results suggest the formation of two distinct dimensions that encode a cognitively meaningful structure of numerical representations. Crucially, the two dimensions were poorly differentiated prior to numerosity training. Further analysis of neuronal subtypes revealed that improved differentiation in layer IPS representations was strongly driven by newly trained and switching neurons (Fig. 8B). Crucially, both the magnitude and mid-point anchoring dimensions are significantly strengthened with numerosity training in layer IPS (Fig. 9). Only the use of the mid-point 5 as an anchor resulted in a linear monotonic increase in this dimension, that is symmetrical for increasing and decreasing distances from the anchor point (Supplementary Fig. S7).

Additional analyses revealed that neural similarity in the latent MDS space between each numerosity and all other numerosities reduced significantly after training in layer IPS (Fig. 9C). While the precision of distributed latent representations improves with training, it is not uniform for all numerosities. Notably, training improved the precision for end-points 1 and 9 via improved differentiation in both dimensions. In contrast, the precision of the mid-point anchor was stronger under dimension 2, but weaker under dimension 1. This result suggests that the salient characteristics of stimuli that contribute to fidelity of population representations, and therefore numerosity identification, may vary depending on where they lie within the overall structure of trained numerical stimuli.

Because MDS encodes ordered structure of stimuli space, one interpretation of the latent representations is that it forms the basis for a mental number line. Crucially, MDS dimensions can be decoded to reveal the distance between consecutive input numerosities in latent representational space (Fig. 9D). MDS representations also clearly reveal how the latent number line structure evolves with numerosity training. Importantly, decoding the latent dimensions allowed us to construct a number line for each dimension (Fig. 9D). Our analyses revealed that prior to training, the latent number line mapped on both MDS dimensions is a logarithmic or non-cyclic power law shaped function^31^. However, after training, this profile changes to a linear one on the first MDS dimension, and to a one-cycle function with a middle reference point, in the second MDS dimension. Notably this profile is in line with developmental changes seen in children who move from logarithmic to one-cycle to linear mental number lines^31,42,57–59^.

Together, these results point to the emergence of an internal structure for numerosity in layer IPS beyond just the observed magnitude representations. Our findings suggest that constructing numbers involves a sense of absolute magnitude as well as relative magnitude^60,61^, and provides a new mechanistic quantitative model of how a two-dimensional structure emerges with learning. This interpretation is consistent with studies of visual object perception in which layer IPS has been shown to capture untangled manifolds of the input representations^62^. Further, our results reveal that creation of this internal structure is dependent on the New+Switch neurons, rather than P-SPONs.

Our study has important implications for understanding the development of cognitively meaningful number sense and learning of numerosity representations in children. Semantically meaningful number sense develops with domain-specific training when the network learns mappings between non-symbolic and symbolic representations of quantity^2,15,29^. Our findings suggest that numerosity representations are primarily encoded in newly trained and switching neurons, rather than P-SPONs, leading to new cognitive representations. Furthermore, we found that distributed and cognitive meaningful representations emerge primarily in higher layers of the visual processing hierarchy, rather than lower perceptual layers V1 and V2. This reflects cognitive constructed representations in the higher layers, and represents a possible candidate mechanism for how individual differences in number sense might develop in children without differences in perceptual capabilities. These findings are in line with the theoretical views which posit that the development of numerical representations involves mapping numerical symbols onto non-symbolic quantities^2,12^. Crucially, our findings demonstrate that this mapping does not depend on the robustness of pre-existing number sensitive neurons.

Developmentally, one of the most important findings of our study was the emergence of latent population-level representations which not only code for absolute magnitude, but also for a relative magnitude characterized by the creation of mid-point anchors (Figs. 8B, C and 9B).

Our findings demonstrate that midpoint anchoring is related to a one cycle number line that emerges during development from early logarithmic or power law shaped number line representations. Our pre-trained network showed a logarithmic or power law function shaped number line, similar to those seen in infants and younger children^31^. With numerosity training we observed the emergence of a one cycle number line similar to what is often observed in children prior to a final shift to linear-like number line representations. Following numerosity training, the latent dimensions of distributed population-level coding captured both one-cycle and linear latent number representations, revealing a mechanism by which this shift might occur developmentally with learning.

Behavioral studies in children have demonstrated a positive correlation between age and accuracy in numerical estimation, which is linked to the number of reference points utilized by the child^63^ and an understanding of the structure of the number line^42,64^. A child with limited comprehension of the numerical range in question may only use the lower endpoint value, treating the task as an open-ended magnitude judgment rather than a proportion judgment^65^. This approach produces estimates well described by an unbounded power function. In contrast, a child who appropriately references both the lower and upper endpoint values on the number line would generate a pattern of over- and underestimation predicted by a one-cycle version of the proportional model, with lower errors at the two endpoints. When a child infers a third reference point at the midpoint of the line, a cyclical pattern of over- and underestimation corresponding to two-cycles arises, with lower errors at all three reference points^42,66^. Across each developmental progression, there is a notable increase in overall accuracy, leading to a developmental shift from a logarithmic or power law shaped function to cyclic to linear estimation^42,65,67^.

Our model uncovered a similar structure characterized by the emergence of mid-point anchors which contribute to increased accuracy with learning. Crucially, we found that accuracy was strongly related to the distance between numerosities in the two-dimensional MDS representations. These distances were much stronger when New+Switch neurons were considered rather than P-SPONs, and when any form of sensitivity to numerosity was considered rather than neurons that were selectively sensitive to numerosity (Fig. 9). On the other hand, purely numerosity selective neurons and P-SPONs showed the same MDS patterns, indicating that they may be sufficient for learning linearity, but active reorganization of number selectivity, and joint selectivity between numbers and orthogonal features, may be required to improve accuracy, thus providing a potential mechanistic distinction between these two aspects.

The results presented here also raise important questions for future studies on learning of numerosity representations. First, progressive training of numbers from small to large, and unsupervised learning of numerosity, can provide further insight into the formation of number sensitive neurons. Second, further studies are needed to determine the mechanisms at the neural and connectivity levels, by which convolution neural networks develop numerosity tuning. Finally, further studies are needed to investigate the effects of counting and arithmetic principles on the development of number sense^19,20^ in DNN models.

To summarize, we implemented in-silico experiments in a biologically inspired nDNN network architecture comprising V1, V2, V3, and IPS layers which model the visual dorsal stream^15,26,27^. We used it to investigate the dynamic evolution of numerosity with an explicit numerosity training paradigm designed to probe mapping between non-symbolic and symbolic representations of quantity, mimicking learning processes experienced by children. We found that learning completely reorganizes neuronal and population level representations of quantity. Moreover, spontaneous numerosity neurons that emerge from domain general object recognition training were not critical to the formation of subsequent number representations. Multiple neuronal characteristics, including preferred numerosities, number sensitivity, tuning acuity, stability, selectivity, and numerical distance effects, as well as distributed population-level neural representational similarity were significantly altered with numerosity training, especially in higher layers V3 and IPS. Multidimensional scaling revealed distinct latent distributed neuronal representations, capturing both absolute and relative aspects of numerosity, including mid-point anchoring. These learnt representations underlie logarithmic to cyclic and linear mental number lines that are characteristic of number sense development in humans. More generally, our findings suggest that spontaneous neurons arising from non-numerosity related visual experiences do not form an essential foundation for numerical cognition. Domain-specific training alters neuronal response characteristics and builds new latent representations that support the development of number sense. These results provide strong evidence against the localist view which posits that number representations are maintained by a group of number selective neurons and argue for a more distributed representation of numerical quantity.

## Methods

### Experimental design

The current study examined the change in neuronal and population level properties that occurred with developmentally relevant numerosity training within a biologically inspired deep learning network that was pre-trained on visual object recognition. The objectives were to investigate the resulting reorganization of the network, assess the relevance of number neurons that emerge spontaneously based on domain general object recognition training to the subsequent development of number sense, investigate the changes in properties of individual neurons as well as changes in distributed population representations, examine the latent structure of distributed population-level neural representations, and identify neuronal tuning and distributed population-level representational features that predict network accuracy. The numerosity training paradigm was based on developmentally relevant numerosity mapping of non-symbolic to symbolic quantities. Details of the model architecture and training stimuli and procedures are described in the respective sub-sections below.

### nDNN model

Our nDNN model consisted of four layers corresponding to V1, V2, V3, and IPS (Fig. 1), key nodes in dorsal visual information processing pathways important for numerical cognition^15,26,27^. Model parameters and architecture were adapted from CORnet-S, a biologically plausible model of the visual pathway^32^, designed to maximize Brain-Score^25^. In the nDNN, V1 and V2 (with recurrent connections) form part of both dorsal and ventral pathways, while V3 and IPS incorporate recurrent connections that allow for flexibility in network tuning^23,32,68,69^. Thus, without loss of generalization, the model parameters offer the most biologically plausible model yet of the dorsal visual pathway. A key goal of the study was to therefore determine whether layer IPS in our nDNN model resembles neuronal tuning properties and functional brain imaging responses observed in humans and non-human primates. Unless otherwise mentioned, all analysis was carried out on the last recursive step of each layer. See Supplementary Methods for details of the architecture. To train the network, first, we initialized it such that it was pre-trained on ImageNet, and then we used the ADAM^70^ optimizer to solve a numerosity task for 50 epochs with default PyTorch parameters (i.e. learning rate 0.001, ${\beta }_{1}=0.9$, ${\beta }_{2}=0.999$, and $\epsilon={10}^{-8}$). For control analyses (Fig. 10), we alternatively used two other optimizers: Stochastic Gradient Descent (SGD) with learning rate 0.001, the Root Mean Squared Propagation (RMSprop) with learning rate 0.001 and default PyTorch parameters (i.e., $\alpha=0.99$, $\epsilon={10}^{-8}$). We based the pre-trained model on the author implementation of CORNet-S in the PyTorch framework, and their pre-trained weights provided in https://github.com/dicarlolab/CORnet.

### Numerosity task and stimuli

In this study, the numerosity task involved associating an image containing dots (the stimulus) to the number of dots it contains, producing a symbolic classification of the number of dots (Fig. 1). We created a visual image dataset containing non-symbolic enumeration data of 1 to 9 randomly colored dots (the same color is used for all dots across an image) on randomly colored backgrounds. We sampled the images to match the input size of network, that is, all images have 3 RGB color channels and are of size 224 × 224. All the images were sampled with a target total area ${TA}$ and a target convex hull area ${CHA}$ in eight different conditions: 2 size conditions × 2 total area conditions × 2 convex hull area conditions. These conditions were balanced for correlations between total dot area and dot size versus numerosity, as well as between convex hull area and dot density versus numerosity, with a factorial design (see Supplementary Methods for details) based on recent advances proposed in human behavioral studies^33,34^. Within each condition, the configurations are parameterized (see Supplementary Methods for details), so that in total, the training dataset contains 8 conditions × 50 parameter sets × 9 numerosities  × 10 samples = 36000 images, and the test dataset contains 8 conditions × 50 parameter sets × 9 numerosities × 2 samples = 7200 images. A set of sample stimuli for numerosities 3, 6, and 9 across the eight different conditions are shown in Supplementary Fig. S10. The resulting patterns of correlations across conditions are summarized in Supplementary Table S11.

### Ablation analysis procedures

We performed the ablation analysis at each epoch on two subsets of IPS neurons: (i) ablating P-SPONs identified at each epoch, and (ii) ablating all neurons except the P-SPONs identified at each epoch. Ablation was implemented by removing the connection between a particular neuron and the neurons it connects to in the next layer.

### Statistical analysis

The current study used the following analytical approaches: (i) identification and classification of number sensitive neurons; (ii) identification of SPONs and P-SPONS; (iii) measuring neuronal properties including tuning precision, stability, selectivity, numerical distance effect and numerical size effect; (iv) representational similarity analysis at a population level; (v) multidimensional scaling of population level representations; and (vi) measurement of representational drift. These aspects are described in their respective sub-sections below.

### Identification and classification of number sensitive neurons

The neurons in each layer and epoch of training were classified into whether they were number sensitive, similar to those used in previous studies^7,8^. First, we ran a one-way ANOVA analysis, to determine if the activation of a neuron differed significantly between different input numerosities. All neurons that demonstrated a significant (*p* < 0.01) main effect of numerosity in their activation levels were classified as number sensitive, with their preferred numerosity being the input value that provided the highest average activation levels. Next, we ran a two-way ANOVA analysis, to determine if the activation of a neuron differed significantly between different input numerosities, but not between stimulus conditions (there were 8 different conditions, with variations in aspects such as total dot area, convex hull area, or whether dots sizes are fixed or randomized). All neurons that demonstrated a significant (*p* < 0.01) main effect of numerosity but not condition in their activation levels were classified as selectively number sensitive, with their preferred numerosity being the input value that provided the highest average activation levels. Neurons classified as number sensitive by the first method subsumed those identified by the second method. Exclusively non-selective numerosity neurons were those identified by the first method but not the second method. See Supplementary Table S3 for related analyses.

### Identification of SPONs and P-SPONs

SPONs were neurons which the respective ANOVA analysis identified as being number sensitive in the pre-trained network, prior to any numerosity training. P-SPONs for a particular epoch were identified based on whether a SPON neuron after a particular numerosity training epoch was still classified as number sensitive (based on the same ANOVA method) with the same preferred numerosity as it had while pre-trained. Thus P-SPONs post-training were identified by the ANOVA analysis both in the pre-trained network and post-training (after 50 epochs) network, with the same preferred numerosity in both.

### Measuring neuronal properties

#### Tuning curve precision

To determine the tuning properties of number sensitive neurons, we performed the following steps: First, we grouped number sensitive neurons by preferred numerosity (PN). We then measured the average activation across these neurons for each input stimuli value to obtain an average activation curve for each PN group. The average activation curves were normalized by dividing by the maximum activation value, so that the activation for PN was equated to 1, and activation for all other inputs were less than 1. The resulting normalized activation curves for each PN were fit to a Gaussian kernel with mean value equal to the PN, and standard deviation estimated as an optimization problem. The standard deviation was converted to a precision measure (precision = inverse of variance).

#### Stability

The measure of stability of each individual neuron was their relative tolerance for how well they preserved responses across identity preserving transformations^35^. To calculate this, we calculated the average activation curve (average activation for each input value) of each neuron separately under the eight different stimuli-type conditions. Thus, for each neuron, we obtained eight different activation profiles, each corresponding to the same set of input identities (numerosities), but under different identity preserving transformations – here, these transformations were balanced manipulations of the dot size, total dot area, and convex hull area. Stable neurons would have similar activation profiles under these eight different conditions. To measure stability, we used Kendall’s tau, which measures rank order agreement (or the rank correlation coefficient) across conditions. High values of tau indicate that the relative rank ordering of input numerosities is similar across the conditions, and vice versa. Li et al.^36^ showed that rank order preservations under transformations were a crucial neuronal property to support accurate recognition. The variability in neuronal responses across these eight conditions are shown in Supplementary Fig. S11.

#### Selectivity

Number sensitive neurons have a preferred numerosity. An ideal number sensitive neuron should always have a higher activation for any physical representation of the preferred numerosity than for any other numerosity. However, this is not always the case. While preferred numerosities on an average have high activation values, some samples of the preferred numerosities elicit lower activation compared to some samples of other numerosities. Since we had 800 samples of each input numerosities in the testing set, we could perform 64k pairwise comparisons (800 × 800) of the preferred numerosity against each other input value. Selectivity is the proportion of such pairwise comparisons where the number sensitive neuron showed higher activation for the preferred numerosity. An ideal number sensitive neuron would have a maximum selectivity of 1.

#### Neuronal numerical distance effect

Numerical distance effects (NDE) refer to the fact that discriminating between numerosities becomes easier as the distance between (input distance) them grows. At a behavioral level, this means increasing accuracy with increasing input distance. At a neuronal level, this might imply increasing selectivity, or larger differences in neuronal activation, with increasing input distance. To compute this, the pairwise selectivity, and pairwise comparison of activation was grouped by the distance between preferred numerosity and the comparative input numerosity being considered. This results in a measure of selectivity and differential activation as a function of input distance (which can take values from 1 to 8). The average slope of the selectivity versus input distance is reported as the NDE. A neuron with higher NDE thus demonstrates a steeper slope of selectivity versus distance, and can be conceptualized as encoding some degree of cognitive content, specifically, the ordinality of numerosity, and access to a mental number line^38^. Higher NDE implies a lower potential for making errors as input distance increases.

#### Neuronal numerical size effects

Numerical size effects refer to the fact that discriminating between numerosities with the same input distance becomes more difficult as the size of the numerosities increases (e.g., discriminating between 2 and 3 is easier than discriminating between 8 and 9, both having the same input distance of 1). Supplementary Fig. S4 shows the neuronal numerical size effect for each input distance value. The pre-trained network shows minimal numerical size effects, but the numerosity trained network shows strong size effects, with reducing accuracy as numerical size (sum of the inputs) increases.

### Representational similarity analysis between input stimuli

Representational similarity analysis (RSA) identifies the similarity between different input numerosities, measured as a similarity of the distributed representations across a selected group of neurons. To do this, the mean activation value of each neuron is calculated for each input stimuli value from 1–9. Next, for each layer and for epochs 0 (pre-training) and 50 (post-training), the mean activations are normalized by dividing by the highest activation value of any neuron for any input within that layer and epoch. This allows for a meaningful comparison across layers and training epochs. Any neurons which are not activated at all for any input are dropped from the analysis (since they represent zero values across all numerosities, they would incorrectly inflate similarity). We then calculated the Euclidean distance between each pair of input stimuli across each of the following groups of neurons: (a) P-SPONs, (b) New+Switch neurons, and (c) all neurons in the layer. These distances are calculated for each layer pre and post training. The distances are then converted to normalized similarity values (1–distance/(maximum distance)), which yields a representational similarity matrix across pairs of input stimuli, where the maximum distance is the highest distance across all layers and epochs. The normalized distance (distance / maximum distance) between pairs of input stimuli from the RSA analysis are also grouped by difference between input values so as to obtain another measure of the numerical distance effect, measured on a distributed population level encoding, rather than the neuronal level NDE measured previously.

### Multidimensional scaling analysis of distributed neural representations

Multidimensional scaling (MDS) is a method for dimension reduction. In each layer with *n* neurons (*n* is approximately 200k for V1, 100k for V2, 50k for V3, and 25k for IPS), the distributed representations of each numerosity are *n*-dimensional. MDS allows us to effectively reduce the dimensionality of representation of each numerosity, such that the pairwise distances between input numerosities in *n*-dimensional space is approximately retained in the reduced dimensionality space. Classical multidimensional scaling was used to reduce the *n*-dimensional representations to two dimensions. MDS eigenvalues and derived goodness of fit^41^ were used to evaluate the adequacy of the MDS algorithm. See Supplementary Methods for details.

### Measuring representational drift

Neuronal representational drift^43,44^ was measured as one minus the rank correlation coefficient (numerosity order preservation) across consecutive epochs for each neuron. Thus, a high drift indicated that the relative ordering of neuronal activation across input numerosities had changed significantly (when measured for each individual neuron and averaged over all neurons), and a low drift indicated rank order preservation between input numerosities. A similar measure of drift was also computed at a distributed representation level by measuring one minus the rank correlation coefficient based on first two latent MDS dimensions for each layer. Both these measures were applied pairwise on consecutive epochs from 1 to 10, and averaged across these epochs. Epochs 1–10 were included since accuracy levels saturate after about 10 epochs.

### Reporting summary

Further information on research design is available in the Nature Portfolio Reporting Summary linked to this article.

## Supplementary information

Supplementary Information

Reporting Summary

## Data Availability

Source data used in figures are provided with this paper. Data is generated from the neural network modeling based on adaptation of the CORNet-S model within the PyTorch framework (Python: 3.9.12; PyTorch: torch==1.12.1, torchaudio==0.12.1, torchmetrics==0.10.0, torchvision==0.13.1, pytorch-lightning==1.8.0.post1). Links to third-party images and model: Original ImageNet stimuli are found at https://image-net.org/download.php. The pre-trained Cornet network is found at https://s3.amazonaws.com/cornet-models/cornet_s-1d3f7974.pth. The key output data for the pre-trained and post-training epochs are stored at 10.5281/zenodo.7976286. Note that data for every training epoch is too large to be stored on this platform, and any specific additional data may be available from the authors on reasonable request. Note that most of the analysis in the paper relies on the pre-trained and post-training epochs for which data has been shared. Source data are provided with this paper.

## Source data

Source Data

## Code availability

Code to produce the number stimuli images as well as to run the models is available under the repository for this manuscript at https://github.com/scsnl/Mistry_Strock_NatureComm_2023. Data analysis was conducted using MATLAB 9.9 R2020b. Data analysis scripts will be made available via GitHub upon publication at https://github.com/scsnl/Mistry_Strock_NatureComm_2023.
