# Supplementary material for: Learning-induced reorganization of number neurons and emergence of numerical representations in a biologically inspired neural network
Source: Nat Commun. 2023 Jun 29;14:3843. doi: 10.1038/s41467-023-39548-5 (PMC10310708; doi:10.1038/s41467-023-39548-5)
Supplement: Supplementary file 1 — Supplementary Information [file 41467_2023_39548_MOESM1_ESM.pdf]

Supplementary Materials for

Learning-induced reorganization of number neurons and emergence of numerical representations in a biologically-inspired deep neural network

Percy K. Mistry, Anthony Strock, Ruizhe Liu, Griffin Young, Vinod Menon

Corresponding authors:
Percy K. Mistry, Ph.D. & Vinod Menon, Ph.D.
email: percym@stanford.edu; menon@stanford.edu

This PDF file includes:

Supplementary Methods
Supplementary Figures S1 to S11
Supplementary Tables S1 to S11

I. Supplementary Methods

nDNN model architecture

The nDNN model is based on the CORNet-S architecture (**Figure 1**). First, V1 receives as input a 224x224 picture with 3 color channels, that is, a 3x224x224 tensor. V1 transforms this input into a 64x56x56 tensor through a chain of a 7x7 convolution with stride 2 augmenting the number of channels to 64, a 3x3 max pooling with stride 2, and a 3x3 convolution, each of the convolution being followed by batch normalization and a ReLU non-linearity. The output of V1 (V2, V3) is then fed as input to V2 (V3, IPS), which transforms it into a 128x28x28 (resp. 256x14x14, 512x7x7) tensor. V2, V3, and IPS are built with the same building block that resembles a recurrent version of a residual block of the ResNet architecture, which has proven to be one of the best performing models on various benchmark datasets in different domains (He, Zhang, Ren, & Sun, 2016). This building block is recurrent, but only the last output it produces is being fed as input to the next block. At time $t = 0$, the input to the block is the last output produced by the previous block, which is first transformed through a 1x1 convolution increasing two times its number of channels. For the following time steps, the input to the block is directly replaced by the feedback of its own output at the previous time step. Moreover, at each time step, the block transforms its input through a chain of 3 convolutions, each followed by batch normalization and a ReLU non-linearity: (1) a 1x1 convolution increasing four times the number of channels, (2) a 3x3 convolution being performed with stride 2 at time $t = 0$, and with stride 1 at all other times, and (3) a 1x1 convolution decreasing four times the number of channels. Furthermore, a skip connection is added between the input before the last ReLU non-linearity which, at time $t = 0$, adds a 1x1 convolution of the input with stride 2 before passing it through the nonlinearity, and at all other times, simply adds the input of the block before passing it through the nonlinearity. V2 and IPS are running for 2 timesteps, whereas V3 is running for 4 timesteps. Unless otherwise stated, we focus the analysis of neurons in V2, V3 and IPS on their last timestep. Finally, to produce the output of the whole model, the last output from IPS is fed in a simple linear decoder preceded by an adaptative average pool which enforces the input of this linear decoder to be of dimension 512 (i.e., the number of output channels of IPS). In the original work, the output has 1000 dimensions as there are 1000 different classes in ImageNet. In this work, we only changed the output dimension of that last linear decoder to match the number of classes we consider, that is, 9 dimensions for 9 classes representing each of the 9 numerosity we consider. Note that in our model, the resulting receptive fields of the V1, V2, V3, and IPS layers increase from under 1% of the image for V1, from 1% to 3% over recursion for V2, from 6% to 38% over recursion for V3, and from 56% to 100% over recursion for IPS. We note that, similar to previous studies, the findings from this model can inform our understanding of the development of numerosity in humans and primates from a representational perspective. While this is a biologically inspired architecture, there are obvious difference between the exact biological mechanisms involved in the nDNN and the human or animal brain. Thus, we can, with reasonable levels of confidence, make conclusions about how numerosity training leads to massive reorganization and shows little to no dependence on pre-trained representations of numerosity, and the nature of these representations, but we can only hypothesize about the precise biological mechanisms leading to such reorganization and change in representations.

Numerosity stimuli and numerosity training

Numerosity stimuli were created in 8 balanced conditions, 2 size conditions x 2 total area conditions x 2 convex hull area conditions: The size conditions required all dots within an image to be either the same size, or be randomly sampled. The total area conditions balance whether or not the total dot area is correlated with numerosity (with non-correlation forcing a correlation between numerosity and average individual dot size or radius). The convex hull area conditions balance whether or not the convex hull area defined by the dot locations is correlated with numerosity (with non-correlation forcing a correlation between numerosity and mean density of dots occupying the area). This results in balanced conditions, since fixing one of these necessarily introduces correlations in one of the others, and it is important to evaluate the development of number sense across these perceptual variations. The total area conditions are denoted as (1) linearly linked to the number of dots N ($TA = \lambda' N$), or (2) fixed ($TA = \lambda$). Similarly, the convex hull area conditions are denoted as either (1) linearly linked to the number of dots N, ($CHA = \mu' N$) or (2) fixed ($CHA = \mu$). To make λ and μ respectively, the mean target total area and the mean target convex hull area across conditions, we fixed $\lambda' = \frac{2\lambda}{9}$ and $\mu' = \frac{2\mu}{9}$. For each of the eight conditions we considered 50 different parameters (λ, μ). We sampled 12 images per condition, per parameter and per numerosity, with 10 being used in training and 2 for testing.

We used a balanced approach to generate the dot stimuli, similar to that used in developmental and human studies [1,2]. The stimuli were generated for a given a target total dot area TA and convex hull area CHA. We performed the sampling in two steps: first, we sampled the size of individual dots and then their location on the picture. When all dots had the same size, we used $\frac{TA}{N}$ to define the individual area of each dot, otherwise, we uniformly sampled the individual area of the $N - 1$ first dots and defined the area of the last so that the sum of all area equals TA. In both cases we made sure that the radii of all the dots was always between $r_{min} = 2$ and $r_{max} = 50$. We sampled the location of the dots on the picture in three steps: first, we uniformly sampled the location of each dot so that its border was at least at a distance $d = 2$ from the border of the image (without taking into account possible overlapping), then we found an initial configuration that ensured that the dots were not overlapping by iteratively moving the overlapping dots away from each other till there was at least a distance $d = 2$ between their borders, and finally we iteratively moved the dots towards (away from) their center of mass, when the current convex hull area was bigger (smaller) than the target CHA, while still ensuring that dots are not overlapping. To make sure that the radii of all the dots always remains between $r_{min} = 2$ and $r_{max} = 50$ and that the convex hull would cover between 30% and 50% of the image, we sampled λ between $9\pi r_{min}^2$ and πr_{max}^2, and μ between 0.3x224x224 and 0.5x224x224. A set of sample stimuli for numerosities 3, 6, and 9 across the eight different conditions are shown in **Supplementary Figure S10**. The resulting conditions, such that whether numerosity was designed to be correlated to total dot area or dot size (removing one correlation introduced the other), and whether numerosity was designed to be correlated to convex hull area or dot density (removing one correlation introduced the other), are summarized in **Supplementary Table S11**. Note that post-numerosity training, the network experiences forgetting of the visual object classification task.

Analysis to show that key results hold regardless of the method used to classify number sensitive neurons

The primary analysis identifies *selectively* numerosity sensitive neurons (henceforth denoted *N*) using a two-way ANOVA analysis as neurons whose activation showed a main effect for numerosity, but did *not* show a main effect for other stimulus conditions (such as size, area, etc.) *or* an interaction effect between numerosity and other stimulus conditions. We also identified *overall* numerosity neurons based on a one-way ANOVA which classified number sensitive neurons as those whose activation levels showed a main effect for numerosity, without considering whether the activation levels also showed a main or interaction effect for other stimuli conditions. The neurons that were identified in the second method included the exclusively number sensitive neurons as well. The neurons identified by the second analysis but not as exclusively number sensitive neurons are called *conjunctively* sensitive neurons (henceforth denoted as *N+C*), since they showed sensitivity to numerosity as well as other stimulus condition (or interaction between numerosity and condition). The exclusively number sensitive condition represent a constrained definition of number sensitivity, but are consistent with most previous studies. The second analysis without such constraints is more generalizable, especially in a biological context. Both analyses are presented here and show that the key conclusions are not dependent on the method used to identify number sensitive neurons.

We analyzed the training induced reorganization of neurons identified as *selectively* number sensitive (**Supplementary Table S1**), and those identified as *overall* number sensitive (**Supplementary Table S2)**. It can be seen that the constrained definition of number sensitivity reduced the proportion of numerosity sensitive neurons in the IPS to about 6% pre-training and 14% post-training, similar to the levels observed in previous studies that employ such a constrained definition, compared to 97.5% pre-training and 100% post-training for overall number sensitive neurons in the IPS. This constrained definition shows that a large proportion of SPONs drop off (become non-numerosity sensitive in the selective sense) after training (between 55% to 80% across layers), whereas a large proportion of SPONs identified using the overall number sensitivity definition switch numerosities after training (between 25% to 86% across layers). However, the key result that a very small proportion of SPONs remains persistent, that is, the proportion of P-SPONs out of original pre-trained SPONs is very low, remains true in both cases (13% - 34% across layers for overall number sensitive and 3% - 17% across layers for selectively number sensitive neurons). The constrained definition thus shows an even smaller proportion of P-SPONs to original SPONs. Finally, the finding that New+Switch neurons (as compared to P-SPONs) form a very large proportion of the post-training number sensitive neurons also remains true in both cases (65% - 87% for all number sensitive neurons and 85% - 99% for selectively number sensitive neurons). Once again, the constrained analysis shows an even larger contribution of New+Switch to post-training number sensitive neurons.

We also compared key neuronal properties based on *selectively* number sensitive neurons (**Supplementary Table S4)**, and overall number sensitive neurons (**Supplementary Table S5),** and showed that once again the key conclusions hold regardless of the method. The tuning precision, stability, selectivity, and NDE results between the two methods were very similar, and

all the key conclusions hold, including the change of patterns across layers post-training, and significant improvements primarily observed in the IPS.

We also determine how neurons shift between being *selectively* numerosity sensitive and being *conjunctively* sensitive to numerosity *and* other stimuli conditions (i.e., neurons identified under the overall method but not the selective method), as well as whether they switch or retain preferred numerosities (**Supplementary Table S3**). This shows that a large proportion of neurons in V3 (39%) and IPS (69%) switch numerosities after numerosity training but stay conjunctively sensitive to both numerosity and condition. Only a small proportion switch from being conjunctively selective to being exclusively selective for numerosity while retaining their preferred numerosity (approximately 2% to 6% of neurons across layers).

Multidimensional scaling analysis of distributed neural representations

Procedure for MDS: First, we calculated the mean activation value of each neuron for each input stimuli value from 1-9. Next, for each layer and for epochs 0 (pre-training) and 50 (post-training), the mean activations are normalized by dividing by the highest activation value of any neuron for any input within that layer and epoch. Then we computed the Euclidean distance of the normalized neuronal activations between each pair of input numerosities. These Euclidean distances are computed across all neurons in each layer (as well as for specific subsets including P-SPONs and New+Switch neurons). These Euclidean distances between each pair of input values were input into a classical multidimensional scaling algorithm that generates an approximation to these distances in an 8-dimensional space (in which the distances between 9 numerosities can be embedded). The MDS approximation can be generated based on any number of dimensions from 1 to 8, with the use of increasing dimensions generating a better fit of the approximation to the original distance data. The goodness of fit metric is based on calculating the ratio of the sum of eigenvalues based on the chosen number of dimensions to the sum of the eigenvalues across all dimensions generated by the MDS procedure. The value of this goodness of fit measure varies from 0 to 1, with higher values indicating a better fit. The goodness of fit for using 1-dimensional and 2-dimensional approximations are shown in **Supplementary Table S8**.

Interpretation of latent structure: The latent MDS structure has specific implications with respect to the resulting shape of the number line. The precise nature of the arch structure observed in **Figure 8A** can be interpreted as being characteristic of the estimated number line, with a perfect semi-circular U-shape with equal distances between points representing a linear number line response profile, and smaller such semi-circular formations representing linear compressions of the number line. The deviation from the semi-circular shape observed here represents a non-linearity in the latent number line representations. The "horseshoe" form of latent representations is known as the Guttman effect [3,4]. This effect is known to characterize a particular form of pattern in data, specifically, that the data has structure with information about the relative size and the relative degree of similarity between stimuli. Specifically, these patterns often occur when the system has good local knowledge of interpoint distances, but larger distances appear grouped together [5,6]. This is characteristic of the reference points created in human latent number lines, and relatively higher precision of points closer to the reference points. This is also observed in the distance effect, which shows a steeper slope for small input differences, but saturates at larger input difference values.

II. Supplementary Figures

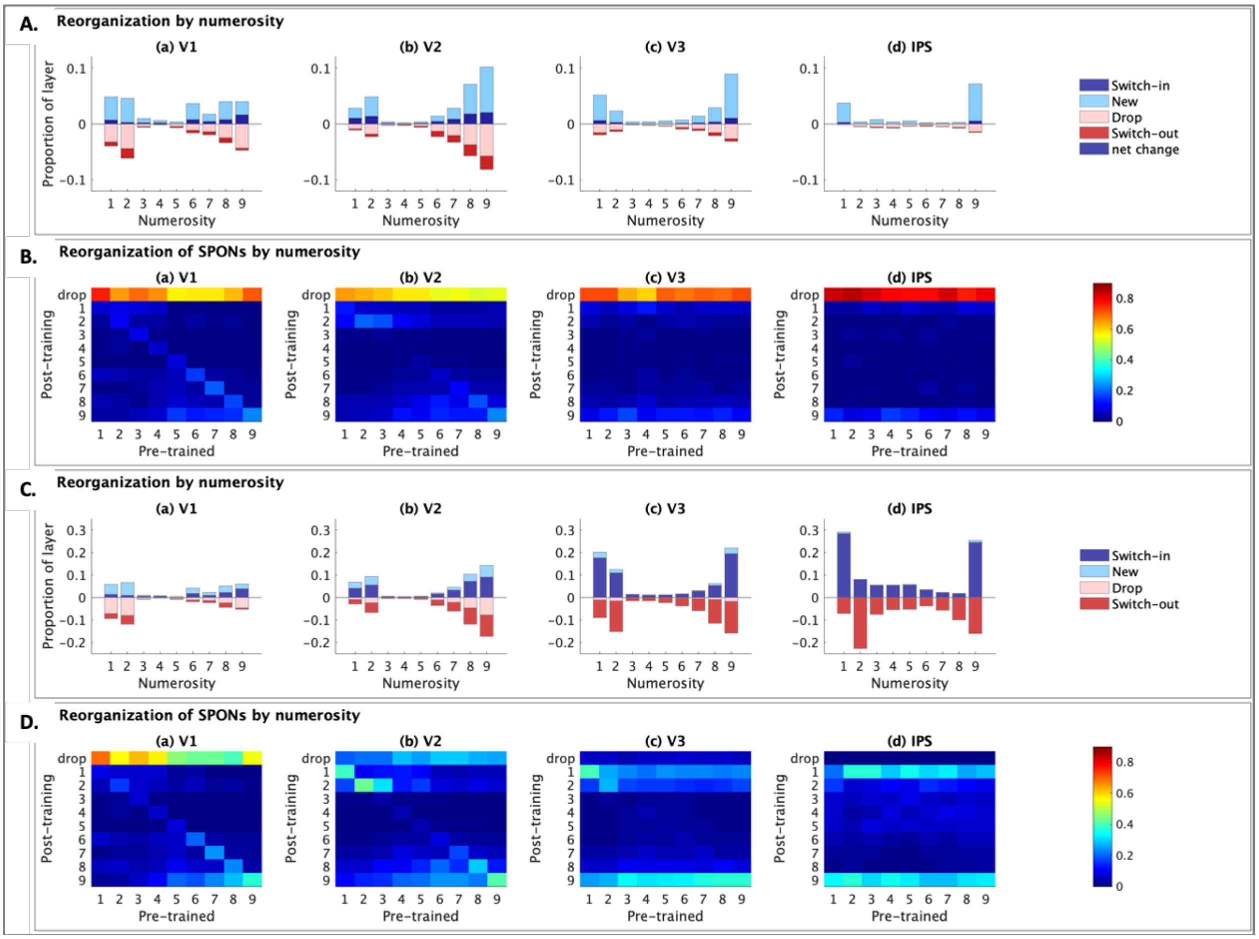

Supplementary Figure S1. (A, C) Reorganization of individual numerosity neurons (A: selective, C: all) between numerosities. **(B, D)** Confusion matrix for switching between numerosities post training (B: selective, D: all). In all panels each layer is shown separately, (a) V1, (b) V2, (c) V3, and (d) IPS.

A.

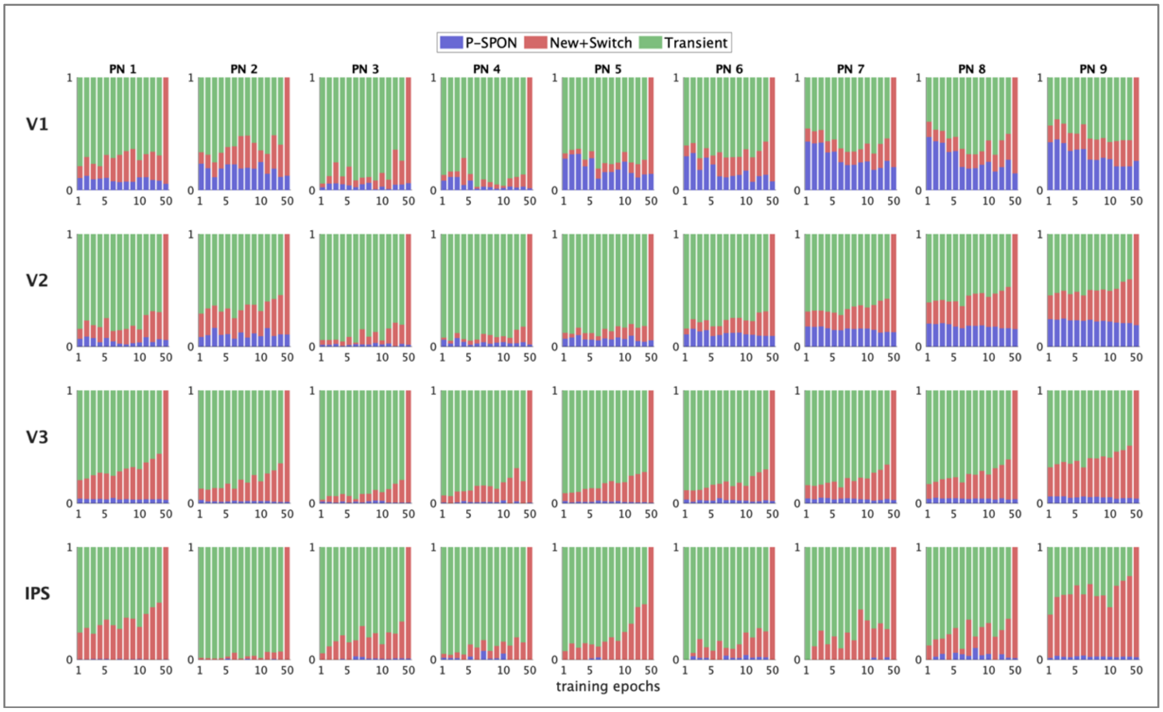

B.

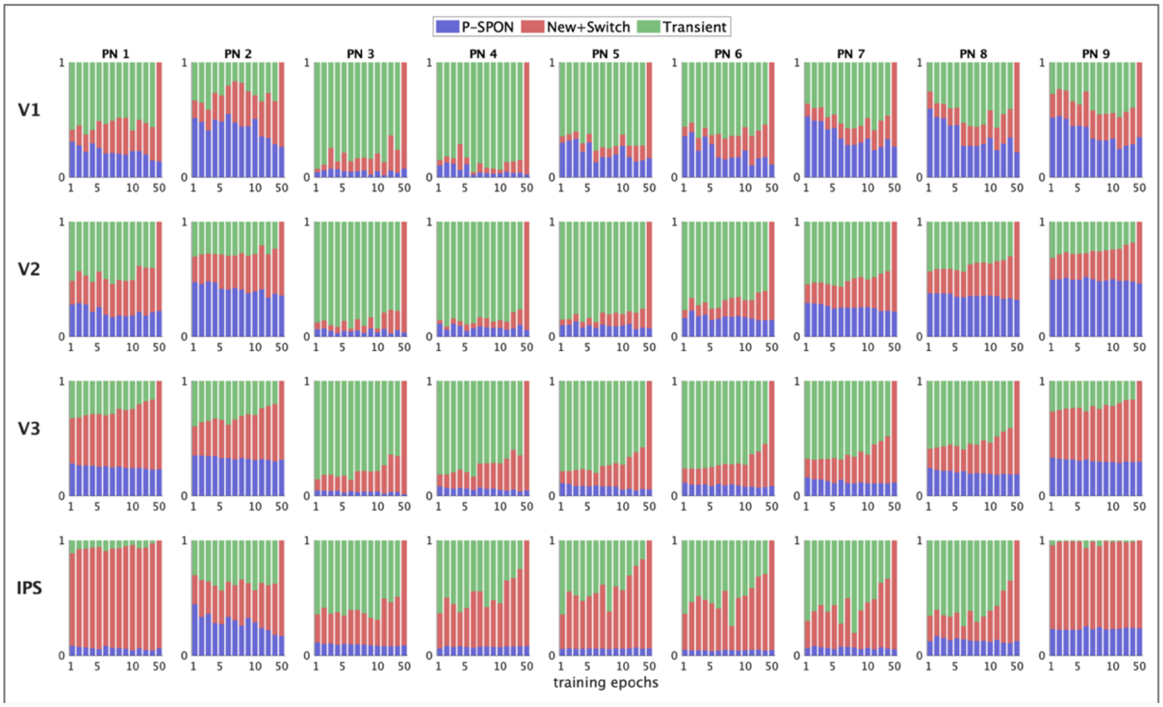

Supplementary Figure S2. Dynamics of neuronal reorganization over training epochs. (A. Selective numerosity neurons, B. All numerosity neurons). The proportion of number-sensitive neurons identified at each training epoch (from 1-10, 20, 30, 40, and 50) for each layer (V1, V2, V3, IPS). P-SPONs (*blue*) were calculated based on evaluation at each particular epoch. Newly-trained (*red*) neurons that were not P-SPONs but maintain the same preferred numerosity

identified during the current epoch at the end of all training (50 epochs). Transient (*green*) are those that are number sensitive in the current training epoch but were neither P-SPONs nor maintained the current preferred numerosity at the end of all training. These are shown separately for each numerosity 1-9. The proportion of new trained neurons (red) gradually increases with training epochs, at the cost of reducing transient neurons. The proportion of transient neurons (green) seems to reduce in higher layers, especially IPS. In the IPS layer, the monotonic numerosities (1 and 9) develop stable neuronal level representations almost immediately, with most of the number sensitive neurons across epochs being newly-trained (red), with very little contribution from transient neurons (green), unlike for numerosities 2-8.

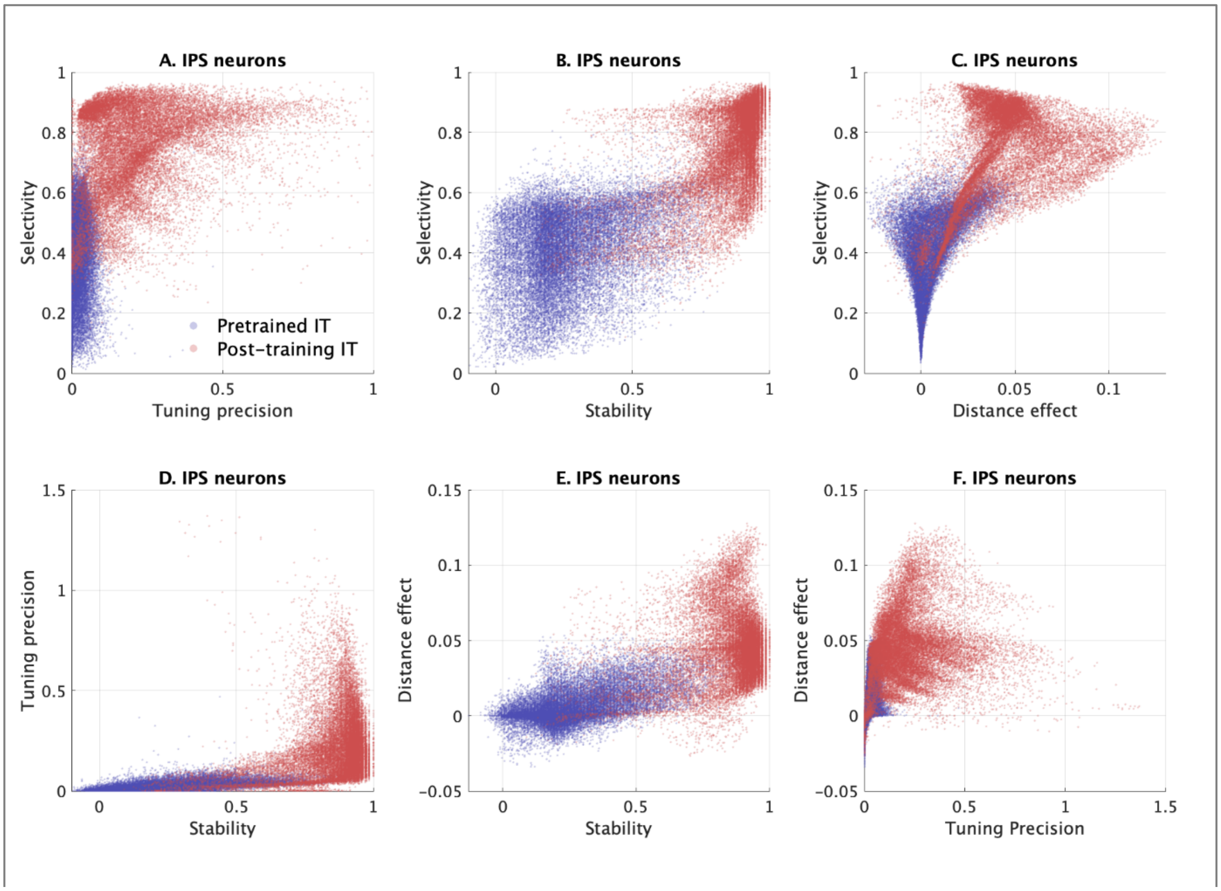

Supplementary Figure S3. **Scatterplots showing the relation between key neuronal properties.** Each dot represents single neuron in the IPS layer, with blue dots showing pretrained and red dots showing post-training neurons. **A.** Neuronal tuning precision versus selectivity. **B.** Stability versus selectivity. **C.** Numerical distance effect (NDE) versus selectivity. **D.** Stability vs tuning precision. **E.** Stability vs NDE. **F.** Tuning precision versus NDE. These plots show that most neuronal properties in the IPS are correlated and that the strength of the correlations increases post-training in all cases except for stability versus NDE.

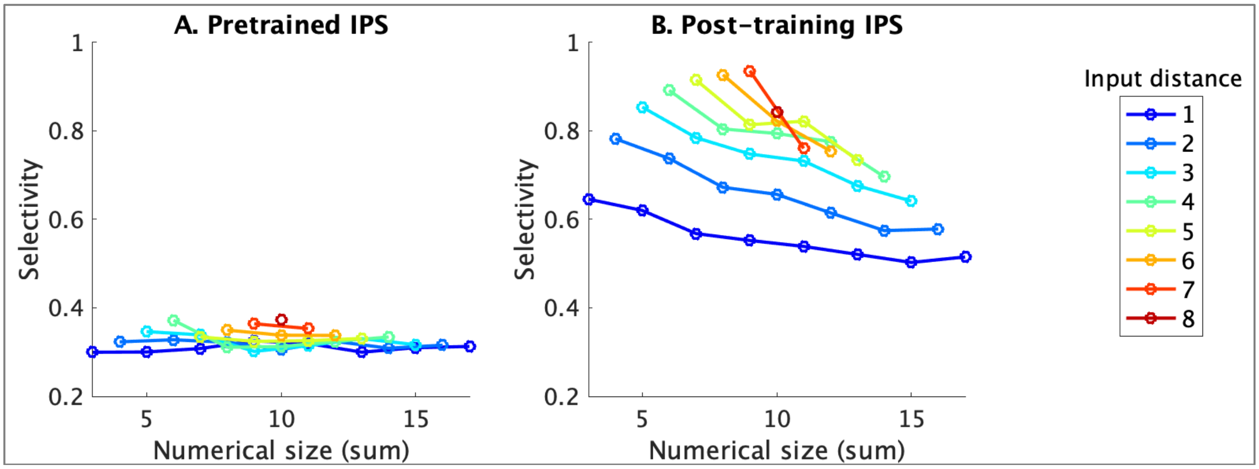

Supplementary Figure S4. **Neuronal selectivity versus numerical size in layer IPS. A.** Pretrained IPS layer **B.** Numerosity trained IPS layer. The plots show the numerical size effect, that is, reducing selectivity as the numerical size (sum) of the inputs increases. The size effect is shown separately for each unique value of the input distance. The size effect is negligible in the pre-trained IPS but post-training IPS shows significant numerical size effects, with increasing size effects for larger input distance values (steeper slopes). This is based on selective number sensitive neurons.

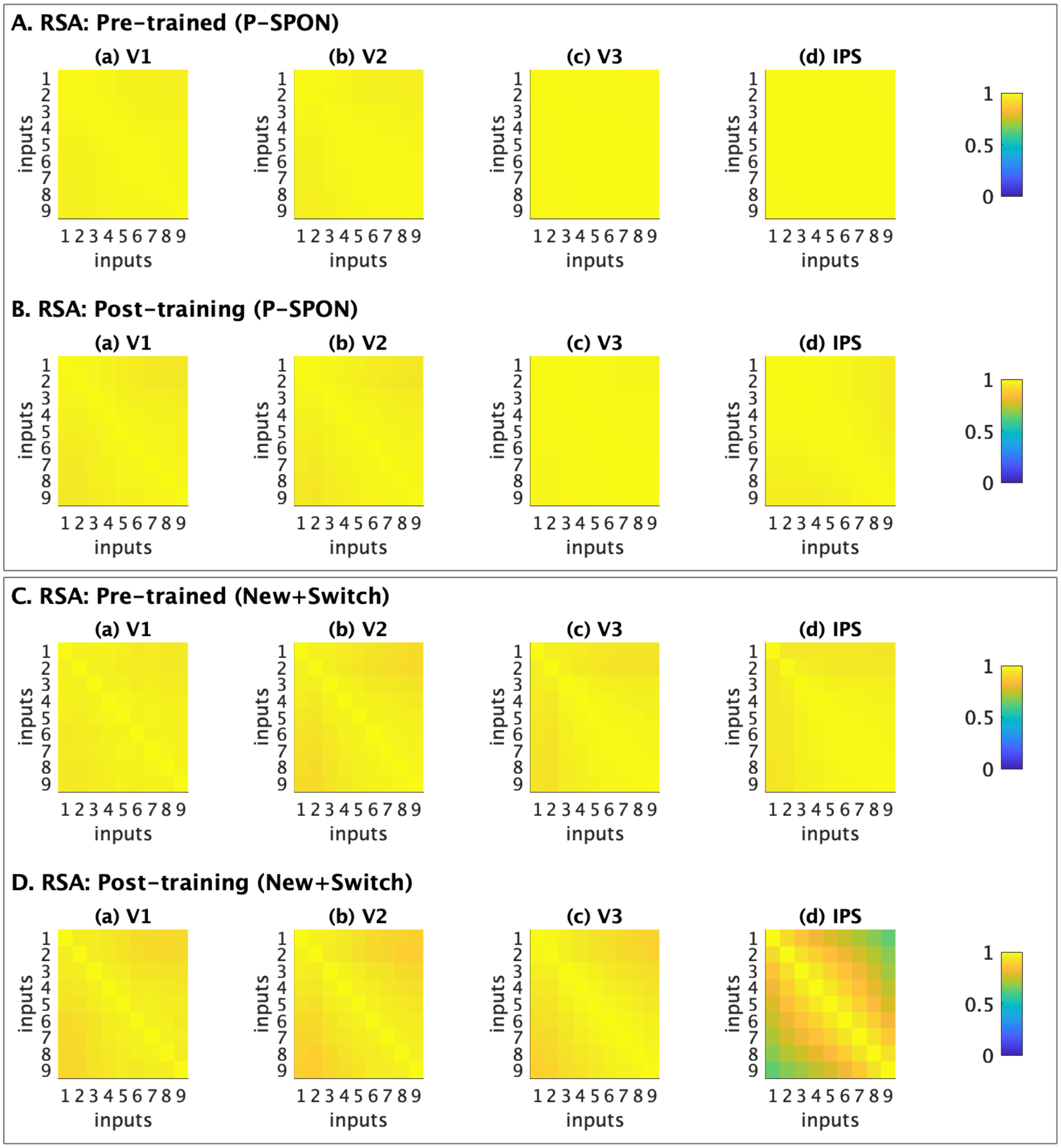

Supplementary Figure S5. Representational similarity analysis based on subsets of neuronal groups in each layer. Subsets based on selective numerosity neurons. A. and **B**. RSA calculated based on pairwise similarity between the mean activation across *P-SPON neurons* in each layer, for each value of the input stimuli, for the pre-trained (A) and post-training (B) networks. **C.** and **D.** RSA calculated based on pairwise similarity between the mean activation across *New+Switch neurons* in each layer, for each value of the input stimuli, for the pre-trained (A) and post-training (B) networks. The pre-trained networks (A, C) show a high degree of similarity across layers. The post-training (B, D) networks show reduced similarity in the IPS layer, with much greater reductions in the similarity calculated using New+Switch IPS neurons (Dd) compared to that calculated using P-SPON IPS neurons (Bd). In all panels each layer is shown separately, (a) V1, (b) V2, (c) V3, and (d) IPS.

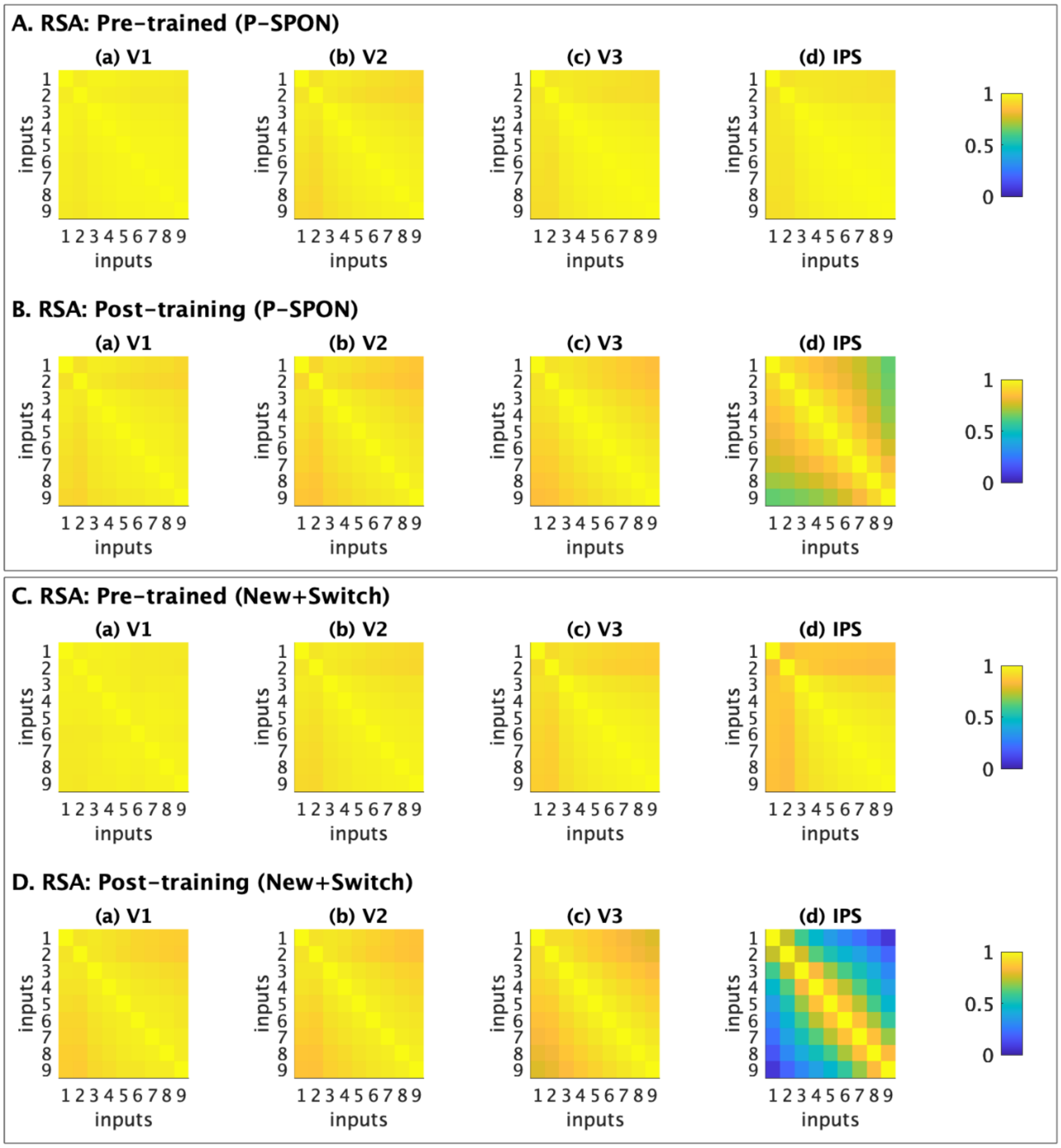

Supplementary Figure S6. Representational similarity analysis based on subsets of neuronal groups in each layer. Subsets based on all numerosity neurons. A. and **B**. RSA calculated based on pairwise similarity between the mean activation across *P-SPON neurons* in each layer, for each value of the input stimuli, for the pre-trained (A) and post-training (B) networks. **C.** and **D.** RSA calculated based on pairwise similarity between the mean activation across *New+Switch neurons* in each layer, for each value of the input stimuli, for the pre-trained (A) and post-training (B) networks. The pre-trained networks (A, C) show a high degree of similarity across layers. The post-training (B, D) networks show reduced similarity in the IPS layer, with much greater reductions in the similarity calculated using New+Switch IPS neurons (Dd) compared to that calculated using P-SPON IPS neurons (Bd). In all panels each layer is shown separately, (a) V1, (b) V2, (c) V3, and (d) IPS.

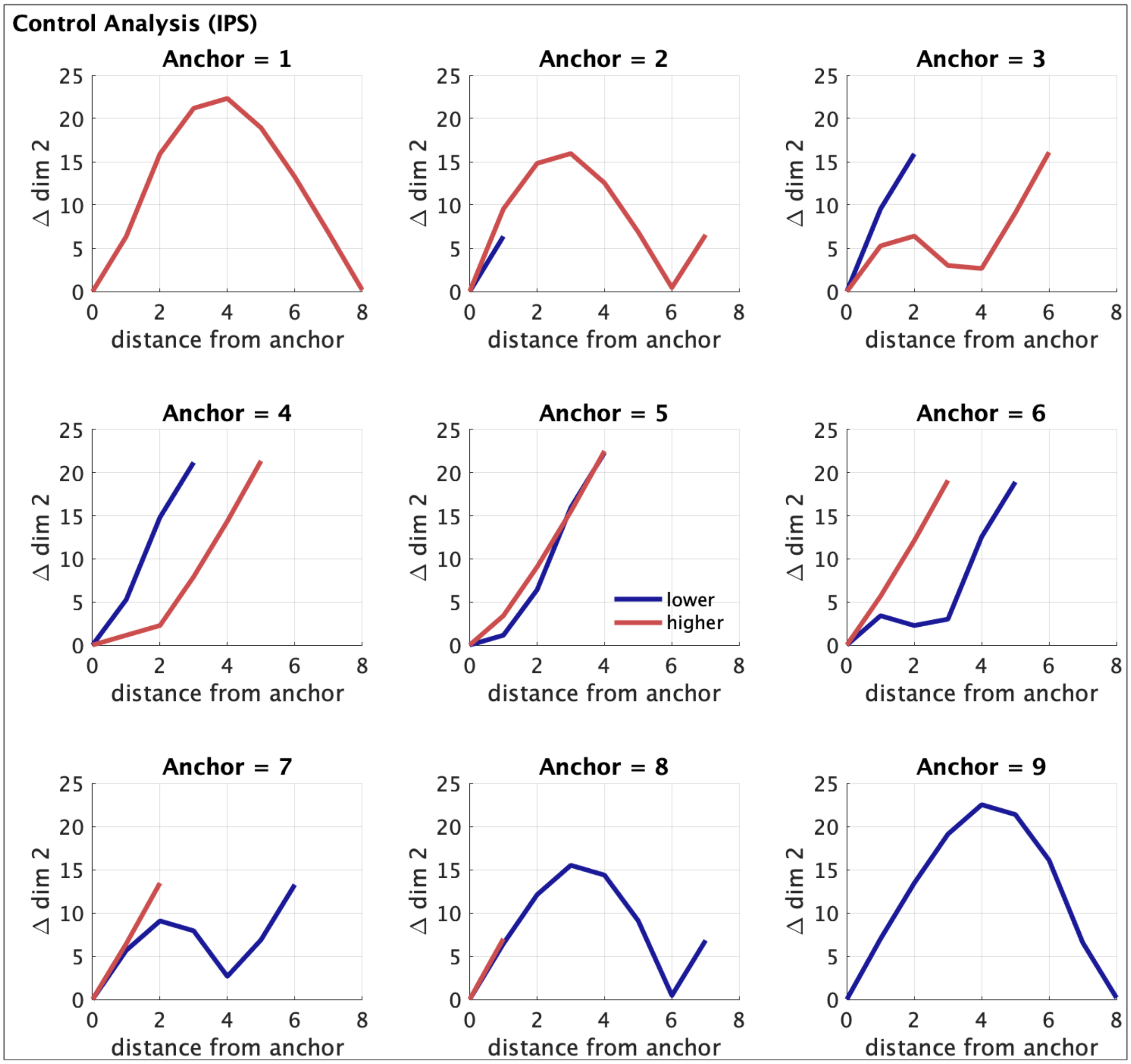

Supplementary Figure S7. Control analysis based on IPS MDS to show the uniqueness of a mid-point anchoring at input numerosity 5. The change in dimension 2 based on distance of the input stimuli with respect to all potential anchors ranging from 1 to 9 is shown (instead of just the mid-point). Only the use of the mid-point 5 as an anchor resulted in a linear monotonic increase in this dimension, that is symmetrical for increasing distances in both directions (increasing (red) and decreasing (blue)), that is, for distances based on input stimuli increasing and decreasing from the anchor point. Using other values (1-4 or 6-9) as anchors does not produce this monotonic and symmetric profile.

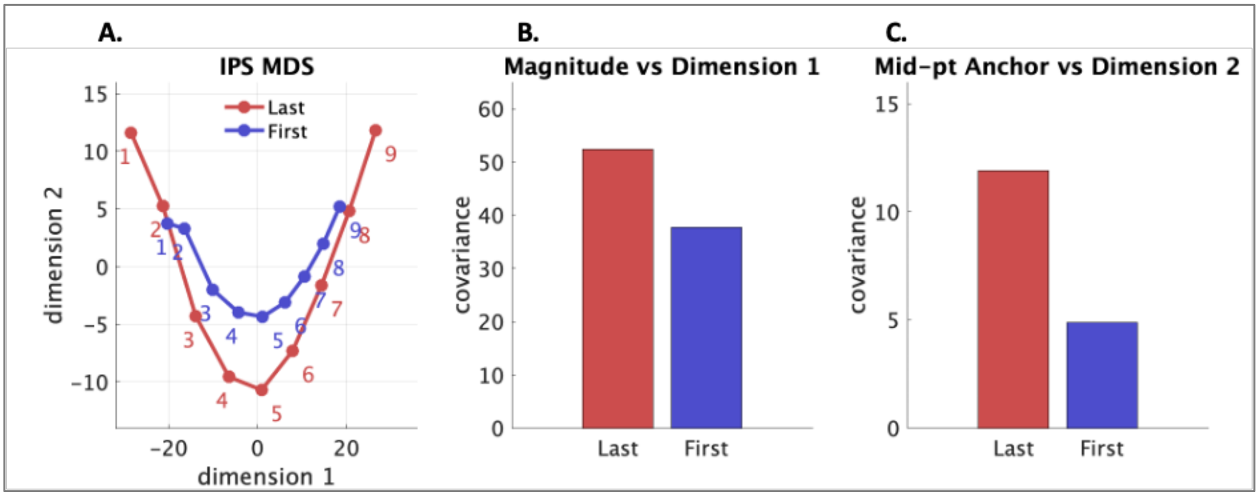

Supplementary Figure S8. Impact of recursion in the nDNN IPS layer. A. MDS representations at the first (pre-recursion) and final (after all recursion) time point within the IPS layer. **B.** The covariance between magnitude and MDS dimension 1 in the IPS layer: recursion increases the covariance. **C.** The covariance between mid-point anchor and MDS dimension 2 in the IPS layer: recursion increases the covariance.

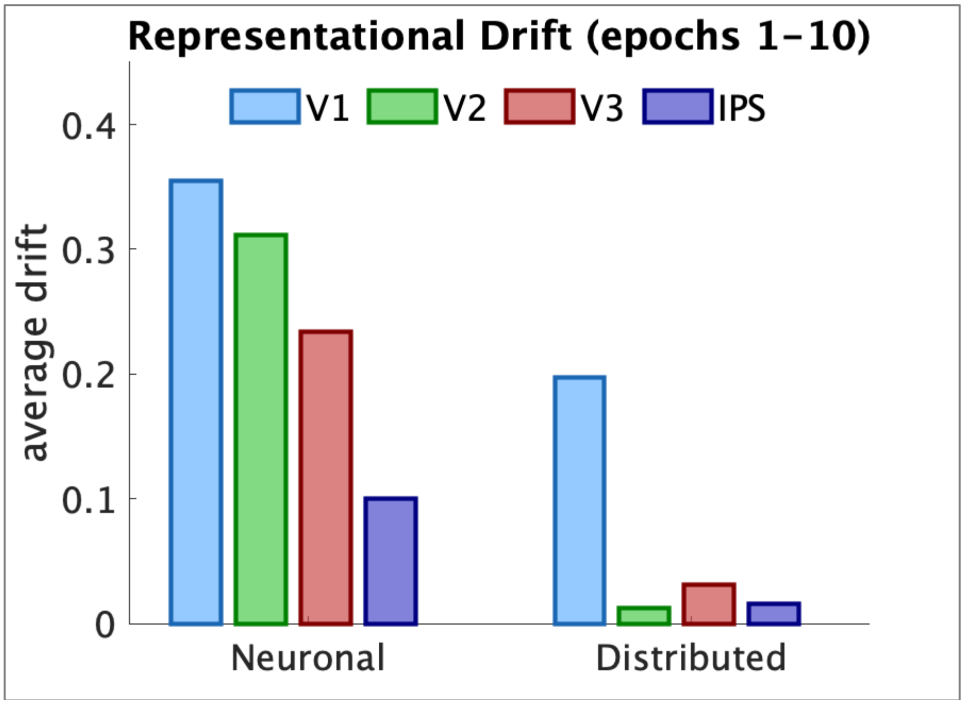

Supplementary Figure S9. Comparison of representation drift measured at the neuronal and distributed level. Representational drift at the neuronal level is measured by calculating one minus the rank correlation coefficient of mean activation for each numerosity between the same neuron on consecutive epochs (i.e., how well does the neuron preserve rank order for activation in response to different numerosities across consecutive training epochs, with high rank order preservation measuring low representational drift, and vice versa). This measure is averaged across the first 10 training epochs, since that is the period when maximal reorganization and improvements in network accuracy occur. This measure is calculated based on the MDS (distributed) representation for each input numerosity (i.e., how well do the MDS dimensions preserve rank order of numerosities across training epochs). The representational drift is higher at a neuronal level than at a distributed level. Further, the representational drift reduces across the information processing hierarchy from V1 to IPS, with low levels of drift in the IPS.

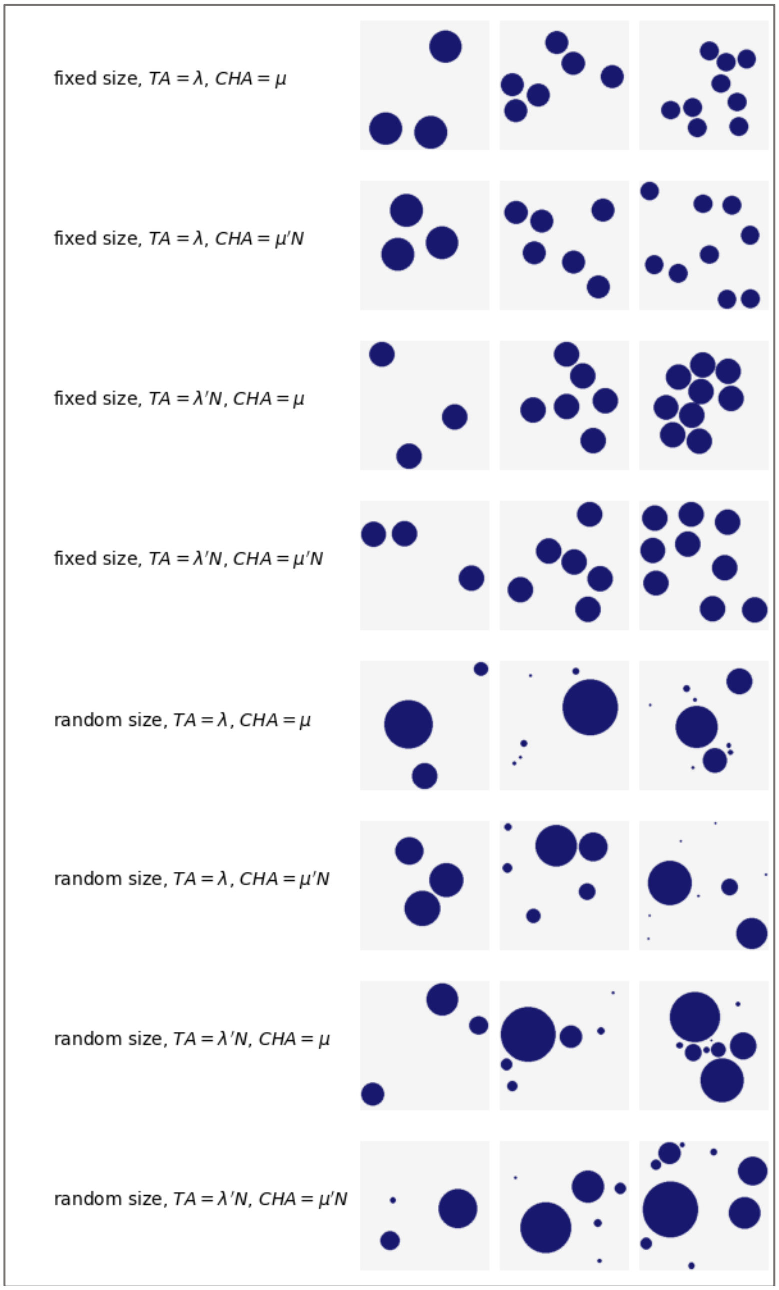

Supplementary Figure S10. Example of non-symbolic input stimuli on which the network is trained and tested. The three columns show numerosities of 3, 6, and 9 respectively. The eight rows illustrate typical examples from the eight different balancing conditions. The first four conditions have fixed dot size within an image, whereas the last four conditions have random dot sizes within an image. The conditions (rows 1,2,5,6) with fixed total area (TA) control for the total area covered by the dots across all values of numerosities, thus higher numerosities will have smaller dots on an average. The conditions (rows 3,4,7,8) with total area proportional to numerosity have equal dot sizes across numerosities on an average, but increasing dot area with increasing numerosity. Similarly, the conditions (rows 1,3,5,7) with fixed convex hull area

(CHA) across numerosities. The convex hull area is formed by the convex area enclosed by drawing an imaginary boundary around all the dots. The conditions (rows 2,4,6,8) with CHA proportional to numerosity has increasing CHA with numerosity.

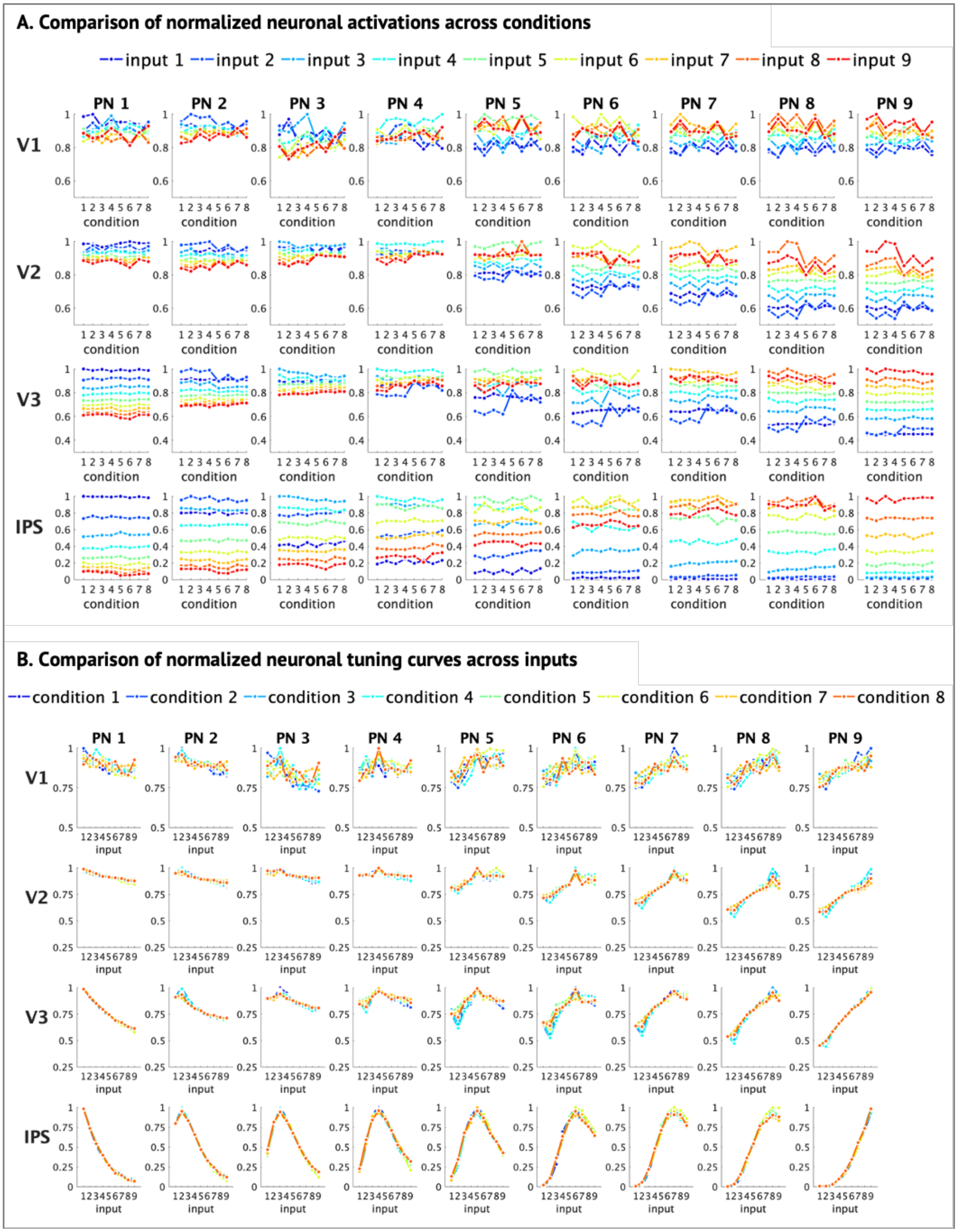

Supplementary Figure S11. Comparison of normalized neuronal activation and tuning curves across conditions. A. Comparison of normalized activation of numerosity neurons across stimuli conditions reveal the underlying basis for relative tolerance post-training. The normalized activation of numerosity selective neurons across the eight stimuli conditions (x-axis) which are based on modifying dot size, total dot area, and convex hull area, to achieve a balanced design, was calculated as activation divided by the maximum activation for each preferred numerosity,

across all inputs and conditions. The rows represent the four layers V1, V2, V3, and IPS. Each colored line represents a particular input numerosity from 1-9. The x-axis in each plot represents the eight different conditions. The y-axis represents normalized activation of numerosity selective neurons by condition for each input stimuli value. Each column of plots represents the average activation for number sensitive neurons of a particular preferred numerosity (columns 1 to 9). Relatively flat lines show high invariance (or tolerance) to conditions, thus showing that the neuronal groups maintain similar activation in the face of identity preserving transformations - the eight different stimuli conditions. Lines that are not flat show lower invariance to the effects of changing conditions. Importantly however, lines that are not flat but do not intersect, still maintain relative rank ordering. Intersecting lines indicate violations of rank ordering of numerosities across conditions, thus showing lower *relative tolerance* for identity preserving transformations. **B.** Similar comparison of normalized activation of numerosity selective neurons across input stimuli. The normalized activation of numerosity selective neurons across the nine input numerosities (x-axis) represents the tuning curves. Each tuning curve is calculated separately for the eight conditions. The rows represent the four layers V1, V2, V3, and IPS. Each colored line represents a particular condition. Each column of plots represents the tuning curves or number sensitive neurons of a particular preferred numerosity (columns 1 to 9). IPS shows near-identical tuning curves across the input values for the 8 conditions, whereas V1 shows noise between the eight conditions.

III. Supplementary Tables

Supplementary Table S1. Reorganization of numerosity preferences of neurons post numerosity training. These data correspond to Figures 3B-E. This is based on *selective* numerosity neurons, identified by a 2-way ANOVA analysis, and including only the neurons that are sensitive to numerosity but not to stimulus condition (that is, the neurons do not show differences between some of the eight conditions that vary in terms of dot area, dot size, and convex hull area). Note that this method identifies only a small proportion of neurons are being number sensitive, similar to previous studies that have employed a similar classification method, with reducing numerosity neurons as we move up the layers from V1 to IPS. Based on this method, a large proportion of SPONs drop because with training, they become sensitive to condition as well as numerosity, even though training is limited to numerosity training.

***Selective* number sensitive neurons**		**V1**	**V2**	**V3**	**IPS**
Numerosity neurons % Layer	pre	27.4%	29.2%	12.6%	6.2%
	post	28.7%	35.0%	23.6%	14.0%
P-SPON % SPON	post	14.5%	17.2%	6.1%	2.9%
Drop % SPON	post	66.4%	55.6%	71.3%	79.9%
Switch % SPON	post	19.1%	27.1%	22.6%	17.2%
Remain % non-SPON	post	73.2%	68.9%	77.1%	86.4%
New numerosity % non-SPON	post	26.8%	31.1%	22.9%	13.6%
P-SPON % Number Sensitive	post	13.8%	14.4%	3.2%	1.3%
New+Switch % Number Sensitive	post	86.2%	85.6%	96.8%	98.7%

Supplementary Table S2. Reorganization of numerosity preferences of neurons post numerosity training. This is based on *all* numerosity neurons, identified by a 1-way ANOVA analysis, regardless of whether the neurons are also sensitive to other stimulus conditions. These data correspond to Figures 3G-J. This shows that a larger proportion of neurons in IPS develop conjunctive sensitivity to both numerosity and condition. The key conclusions, that P-SPONs form a small proportion of SPONs, and that New+Switch neurons form a significantly large proportion of the post-training number sensitive neurons, hold regardless of the method used to identify numerosity neurons.

***Overall* number sensitive neurons**		**V1**	**V2**	**V3**	**IPS**
Numerosity neurons % Layer	pre	47.3%	76.6%	90.4%	97.5%
	post	41.0%	73.9%	91.9%	100.0%
P-SPON % SPON	post	19.7%	33.5%	25.5%	13.6%
Drop % SPON	post	55.0%	25.5%	7.0%	0.0%
Switch % SPON	post	25.4%	41.0%	67.5%	86.4%
Remain % non-SPON	post	62.6%	27.9%	18.9%	0.0%
New numerosity % non-SPON	post	37.4%	72.1%	81.2%	100.0%
P-SPON % Number Sensitive	post	22.7%	34.7%	25.1%	13.3%
New+Switch % Number Sensitive	post	77.4%	65.3%	74.9%	86.7%

Supplementary Table S3. Reorganization between selective and non-selective numerosity neurons. These data correspond to plots 3K(a-e). Extended details of reorganization based on methods used for identifying number sensitivity. In contrast to previous studies that have used a 2-way ANOVA to select neurons that are exclusively sensitive only to numerosity (denoted *N* in this table), to the exclusion of being sensitive to any other perceptual features (e.g., to area), we have used a 1-way ANOVA to select neurons that showed sensitivity to numerosity, regardless of whether they also showed sensitivity to other perceptual features (manipulated in the different stimuli conditions). Thus, all neurons identified using the first method will be a subset of the second. We use *N+C* to denote neurons that are identified as number-sensitive as per the second method, but *not* as per the first, that is, those that specifically display some form of conjunctive sensitivity to both numerosity and stimuli condition. This table shows neuronal reorganization in terms of shifts that may occur between these categories. For example, a neuron that was selectively sensitive to numerosity pre-trained (*N*), and changed to conjunctive sensitivity (*N+C*: sensitive to both numerosity and condition) post-training, while retaining its preferred numerosity (*same*), would fall under the fourth row in the table. The table shows the proportion of neurons in each layer that fall under each category. The rows show the different possible transitions of neurons from pretrained to post-training. A large proportion of neurons in V3 and IPS switch numerosities and stay conjunctively sensitive to both numerosity and condition. Proportions greater than 10% are highlighted in bold.

Pre-trained	Post-training	Preferred Numerosity	V1	V2	V3	IPS
N	N	Same	4.0%	5.0%	0.8%	0.2%
N+C	N+C	Same	2.6%	**11.9%**	**16.2%**	**10.4%**
N+C	N	Same	1.6%	6.1%	4.5%	2.0%
N	N+C	Same	1.2%	2.7%	1.6%	0.7%
N	N	Switch	5.2%	7.9%	2.8%	1.1%
N+C	N+C	Switch	1.8%	**11.0%**	**39.2%**	**68.7%**
N+C	N	Switch	3.5%	8.2%	**13.2%**	**10.2%**
N	N+C	Switch	1.5%	4.4%	5.8%	4.3%
N	Not NS	-	**15.5%**	9.2%	1.5%	0.0%
N+C	Not NS	-	**10.5%**	**10.4%**	4.8%	0.0%
Not NS	N	-	**14.4%**	7.8%	2.3%	0.5%
Not NS	N+C	-	5.3%	9.0%	5.5%	2.0%
Not NS	Not NS	-	**32.9%**	6.5%	1.8%	0.0%

Supplementary Table S4. Change in key neuronal properties from pre-trained to post numerosity training, for *selective* numerosity neurons. These data correspond to Figures 4D-F.

**Neuronal properties of *selective* number sensitive neurons**		**V1**	**V2**	**V3**	**IPS**
Tuning Precision	pre	0.0041	0.0106	0.0128	0.0265
	post	0.0057	0.0132	0.0214	**0.1895**
Stability	pre	0.1864	0.2705	0.3121	0.3437
	post	0.1737	0.3002	0.4414	**0.9139**
Selectivity	pre	0.5090	0.3944	0.4359	0.3265
	post	0.4773	0.3787	0.4010	**0.7326**
Numerical Distance Effect (NDE)	pre	0.0029	0.0033	0.0069	0.0062
	post	0.0036	0.0035	0.0131	**0.0408**

Supplementary Table S5. Change in key neuronal properties from pre-trained to post numerosity training for all numerosity neurons. The tuning precision, stability, selectivity, and NDE calculated are very similar to those calculated for only selective numerosity neurons shown in supplementary table S4.

**Neuronal properties of *all* number sensitive neurons**		**V1**	**V2**	**V3**	**IPS**
Tuning precision	pre	0.0038	0.0126	0.0128	0.0195
	post	0.0055	0.0128	0.0199	**0.1761**
Stability	pre	0.1839	0.2594	0.2990	0.2784
	post	0.1762	0.2786	0.4649	**0.8515**
Selectivity	pre	0.5159	0.3952	0.5005	0.4166
	post	0.4886	0.3905	0.5012	**0.7466**
Numerical Distance Effect (NDE)	pre	0.0029	0.0035	0.0096	0.0067
	post	0.0037	0.0036	0.0166	**0.0449**

Supplementary Table S6. Correlation between neuronal properties in the IPS layer pre and post training (* = p < 0.0001). Conjunctive numerosity neurons are those that are identified as number sensitive neurons (overall) *but not as selectively* number sensitive neurons. Pearson correlation coefficients are reported and significance is corrected for multiple comparisons (conjunctive n = 22896 (pre) and 21576 (post); selective n = 1561 (pre) and 3512 (post); all n = 24456 (pre) and 25088 (post)).

	Conjunctive numerosity		Selective Numerosity		All Numerosity	
**Correlation coefficient**	**Pre-trained**	**Post training**	**Pre-trained**	**Post training**	**Pre-trained**	**Post training**
Selectivity vs Precision	-0.040*	0.259*	-0.197*	-0.074*	-0.043*	0.233*
Selectivity vs Stability	0.309*	0.597*	0.488*	0.575*	0.296*	0.572*
Selectivity vs NDE	0.461*	0.366*	0.658*	0.280*	0.463*	0.359*
Stability vs Precision	0.113*	0.262*	0.350*	0.131*	0.115*	0.249*
Stability vs NDE	0.561*	0.224*	0.587*	0.020	0.556*	0.193*
Precision vs NDE	0.082*	0.160*	0.253*	-0.324*	0.083*	0.127*

Supplementary Table S7. Average slope of (1-average RSA similarity) versus input distance. This measures the numerical distance effect at a distributed level. Larger values imply stronger distance effects, and larger dissimilarity between the distributed representations of input stimuli with increasing numerosity difference. The IPS layer shows significantly larger slopes post numerosity training.

Average slope for (1-RSA) vs distance		**V1**	**V2**	**V3**	**IPS**
Distributed NDE	pre	0.003	0.0079	0.0110	0.0139
	post	0.011	0.0145	0.0255	**0.1080**

Supplementary Table S8. Goodness of fit for MDS of numerosity representations. These values vary from 0 to 1, with higher values indicating a better fit.

		**V1**	**V2**	**V3**	**IPS**
Approximation to 1 dimension	pre	0.54	0.75	0.76	0.58
	post	0.65	0.77	0.90	0.79
Approximation to 2 dimensions	pre	0.70	0.89	0.93	0.91
	post	0.78	0.89	0.97	0.96

Supplementary Table S9. Control analyses.
Reproduction of **Supplementary Table S4** (pre and post training), then compared to three control analyses (C1: Epoch 1; C2: RMS propagation; C3: Stochastic gradient descent).

**Exclusively number sensitive neurons**		**V1**	**V2**	**V3**	**IPS**
Tuning Precision	pre	0.0041	0.0106	0.0128	0.0265
	post	0.0057	0.0132	0.0214	**0.1895**
	C1	0.0042	0.0110	0.0289	**0.2117**
	C2	0.0058	0.0149	0.0239	**0.1365**
	C3	0.0055	0.0120	0.0197	**0.1020**
Stability	pre	0.1864	0.2705	0.3121	0.3437
	post	0.1737	0.3002	0.4414	**0.9139**
	C1	0.1595	0.2986	0.5436	**0.9183**
	C2	0.1958	0.3277	0.4403	**0.8851**
	C3	0.1987	0.3074	0.4324	**0.8320**
Selectivity	pre	0.5090	0.3944	0.4359	0.3265
	post	0.4773	0.3787	0.4010	**0.7326**
	C1	0.4919	0.4646	0.5052	**0.7986**
	C2	0.4535	0.3807	0.3908	**0.6905**
	C3	0.4607	0.4669	0.4902	**0.6790**
Numerical Distance Effect (NDE)	pre	0.0029	0.0033	0.0069	0.0062
	post	0.0036	0.0035	0.0131	**0.0408**
	C1	0.0028	0.0004	0.0170	**0.0419**
	C2	0.0035	0.0023	0.0089	**0.0357**
	C3	0.0022	0.0042	0.0111	**0.0398**

Supplementary Table S10. Control analyses.
Reproduction of **Supplementary Table S1** (pre and post training), then compared to three control analyses (C1: Epoch 1; C2: RMS propagation; C3: Stochastic gradient descent).

**Exclusively number sensitive neurons**		**V1**	**V2**	**V3**	**IPS**
Numerosity neurons % Layer	pre	27.4%	29.2%	12.6%	6.2%
	post	28.7%	35.0%	23.6%	14.0%
	C1	24.9%	35.6%	15.9%	10.7%
	C2	27.1%	39.9%	24.6%	15.8%
	C3	25.2%	35.3%	13.9%	5.9%
P-SPON % SPON	post	14.5%	17.2%	6.1%	2.9%
	C1	26.5%	22.7%	5.5%	1.2%
	C2	10.4%	18.0%	7.3%	4.5%
	C3	38.9%	36.5%	11.7%	2.5%
Drop % SPON	post	66.4%	55.6%	71.3%	79.9%
	C1	58.7%	51.6%	77.3%	87.6%
	C2	73.5%	50.2%	71.1%	75.0%
	C3	45.6%	38.7%	70.3%	89.8%
Switch % SPON	post	19.1%	27.1%	22.6%	17.2%
	C1	14.8%	25.7%	17.2%	11.1%
	C2	16.1%	31.8%	21.5%	20.5%
	C3	15.5%	24.8%	17.9%	7.7%
P-SPON % Number Sensitive	post	13.8%	14.4%	3.2%	1.3%
	C1	29.1%	18.7%	4.4%	0.7%
	C2	10.5%	13.2%	3.8%	1.8%
	C3	42.2%	30.2%	10.6%	2.7%
New+Switch % Number Sensitive	post	86.2%	85.6%	96.8%	98.7%
	C1	70.9%	81.3%	95.6%	99.3%
	C2	89.5%	86.8%	96.2%	98.2%
	C3	57.8%	69.8%	89.4%	97.3%

Supplementary Table S11.

The table shows what attributes of the non-symbolic stimuli are correlated with numerosity (Y = correlated, N = Not correlated). Note that removing correlations with total area (TA) introduces correlations with dot size, and removing correlations with the convex hull area (CHA) introduces correlations with dot density. The conditions balance out these correlations. The conditions can only be accurately specified for numerosities of 3 and higher.

Condition	Dot size	TA	CHA	Correlation of numerosity with:			
				TA	CHA	Dot Size	Density
1	fixed	λ	μ	N	N	Y	Y
2	fixed	λ	μ′N	N	Y	Y	N
3	fixed	λ′N	μ	Y	N	N	Y
4	fixed	λ′N	μ′N	Y	Y	N	N
5	random	λ	μ	N	N	Y	Y
6	random	λ	μ′N	N	Y	Y	N
7	random	λ′N	μ	Y	N	N	Y
8	random	λ′N	μ′N	Y	Y	N	N

Supplementary References

1 De Marco, D. & Cutini, S. Introducing CUSTOM: A customized, ultraprecise, standardization-oriented, multipurpose algorithm for generating nonsymbolic number stimuli. *Behavior Research Methods* **52**, 1528-1537 (2020).

2 Guillaume, M., Schiltz, C. & Van Rinsveld, A. NASCO: A new method and program to generate dot arrays for non-symbolic number comparison tasks. *Journal of Numerical Cognition* **6**, 129-147 (2020).

3 Camiz, S. The Guttman effect: its interpretation and a new redressing method. *Data Analysis Bulletin* **5**, 7-34 (2005).

4 Morton, J. T. *et al.* Uncovering the horseshoe effect in microbial analyses. *Msystems* **2**, e00166-00116 (2017).

5 Diaconis, P., Goel, S. & Holmes, S. Horseshoes in multidimensional scaling and local kernel methods. *The Annals of Applied Statistics* **2**, 777-807 (2008).

6 Kent, J., Bibby, J. & Mardia, K. *Multivariate analysis*. (Academic Press Amsterdam, 1979).
