# Supplementary material for: Learning-induced reorganization of number neurons and emergence of numerical representations in a biologically inspired neural network
Source: Nat Commun. 2023 Jun 29;14:3843. doi: 10.1038/s41467-023-39548-5 (PMC10310708; doi:10.1038/s41467-023-39548-5)
Supplement: Supplementary file 2 — Reporting Summary [file 41467_2023_39548_MOESM2_ESM.pdf]

nature portfolio

Corresponding author(s): Percy Mistry, Vinod Menon

Last updated by author(s): May 25, 2023

Reporting Summary

Nature Portfolio wishes to improve the reproducibility of the work that we publish. This form provides structure for consistency and transparency in reporting. For further information on Nature Portfolio policies, see our Editorial Policies and the Editorial Policy Checklist.

Statistics

For all statistical analyses, confirm that the following items are present in the figure legend, table legend, main text, or Methods section.

n/a	Confirmed	
☐	☒	The exact sample size (*n*) for each experimental group/condition, given as a discrete number and unit of measurement
☒	☐	A statement on whether measurements were taken from distinct samples or whether the same sample was measured repeatedly
☐	☒	The statistical test(s) used AND whether they are one- or two-sided<br>*Only common tests should be described solely by name; describe more complex techniques in the Methods section.*
☐	☒	A description of all covariates tested
☐	☒	A description of any assumptions or corrections, such as tests of normality and adjustment for multiple comparisons
☐	☒	A full description of the statistical parameters including central tendency (e.g. means) or other basic estimates (e.g. regression coefficient) AND variation (e.g. standard deviation) or associated estimates of uncertainty (e.g. confidence intervals)
☐	☒	For null hypothesis testing, the test statistic (e.g. *F*, *t*, *r*) with confidence intervals, effect sizes, degrees of freedom and *P* value noted<br>*Give P values as exact values whenever suitable.*
☒	☐	For Bayesian analysis, information on the choice of priors and Markov chain Monte Carlo settings
☒	☐	For hierarchical and complex designs, identification of the appropriate level for tests and full reporting of outcomes
☐	☒	Estimates of effect sizes (e.g. Cohen's *d*, Pearson's *r*), indicating how they were calculated

Our web collection on statistics for biologists contains articles on many of the points above.

Software and code

Policy information about availability of computer code

Data collection	Data is generated from the neural network modeling based on adaptation of the CORNet-S model within the PyTorch framework (Python: 3.9.12; PyTorch: torch==1.12.1, torchaudio==0.12.1, torchmetrics==0.10.0, torchvision==0.13.1, pytorch-lightning==1.8.0.post1). Code to produce the number stimuli images as well as to run the models is available under the repository for this manuscript at https://github.com/scsnl/Mistry_Strock_NatureComm_2023.<br>Links to third-party images and model: Original ImageNet stimuli are found at https://image-net.org/download.php. The pre-trained Cornet network is found at https://s3.amazonaws.com/cornet-models/cornet_s-1d3f7974.pth.
Data analysis	Data analysis was conducted using MATLAB 9.9 R2020b. Data analysis scripts will be made available via GitHub upon publication at https://github.com/scsnl/Mistry_Strock_NatureComm_2023.

For manuscripts utilizing custom algorithms or software that are central to the research but not yet described in published literature, software must be made available to editors and reviewers. We strongly encourage code deposition in a community repository (e.g. GitHub). See the Nature Portfolio guidelines for submitting code & software for further information.

Data

Policy information about availability of data

All manuscripts must include a data availability statement. This statement should provide the following information, where applicable:

- Accession codes, unique identifiers, or web links for publicly available datasets
- A description of any restrictions on data availability
- For clinical datasets or third party data, please ensure that the statement adheres to our policy

Data for the figures is included as a source data file. The key output data for the pre-trained and post-training epochs are stored at 10.5281/zenodo.7976287. Note that data for every training epoch is too large to be stored on this platform, and any additional specific data may be available from the authors on reasonable request. Note that most of the analysis in the paper relies on the pre-trained and post-training epochs for which data has been shared.
Links to third-party images and model: Original ImageNet stimuli are found at https://image-net.org/download.php. The pre-trained Cornet network is found at https://s3.amazonaws.com/cornet-models/cornet_s-1d3f7974.pth.

Human research participants

Policy information about studies involving human research participants and Sex and Gender in Research.

Reporting on sex and gender	N/A
Population characteristics	N/A
Recruitment	N/A
Ethics oversight	N/A

Note that full information on the approval of the study protocol must also be provided in the manuscript.

Field-specific reporting

Please select the one below that is the best fit for your research. If you are not sure, read the appropriate sections before making your selection.

☐ Life sciences ☒ Behavioural & social sciences ☐ Ecological, evolutionary & environmental sciences

For a reference copy of the document with all sections, see nature.com/documents/nr-reporting-summary-flat.pdf

Behavioural & social sciences study design

All studies must disclose on these points even when the disclosure is negative.

Study description	Quantitative data analysis based on data generated by a biologically inspired neural network. No data collected from humans or any other life form.
Research sample	Data generated by a biologically inspired neural network. Links to third-party images and model: Original ImageNet stimuli are found at https://image-net.org/download.php. The pre-trained Cornet network is found at https://s3.amazonaws.com/cornet-models/cornet_s-1d3f7974.pth.
Sampling strategy	No traditional data collection involved. Data generated by a biologically inspired neural network,
Data collection	No traditional data collection involved. Data generated by a biologically inspired neural network.
Timing	No traditional data collection involved. Data generated by a biologically inspired neural network.
Data exclusions	No data excluded.
Non-participation	No participants / non-participation involved.
Randomization	No participants / randomization, and no relevant covariates to be controlled.

Reporting for specific materials, systems and methods

We require information from authors about some types of materials, experimental systems and methods used in many studies. Here, indicate whether each material, system or method listed is relevant to your study. If you are not sure if a list item applies to your research, read the appropriate section before selecting a response.

Materials & experimental systems

n/a	Involved in the study
☒	☐ Antibodies
☒	☐ Eukaryotic cell lines
☒	☐ Palaeontology and archaeology
☒	☐ Animals and other organisms
☒	☐ Clinical data
☒	☐ Dual use research of concern

Methods

n/a	Involved in the study
☒	☐ ChIP-seq
☒	☐ Flow cytometry
☒	☐ MRI-based neuroimaging
